# The retaining β-Kdo glycosyltransferase WbbB uses a double-displacement mechanism with an intermediate adduct rearrangement step

**DOI:** 10.1038/s41467-022-33988-1

**Published:** 2022-10-21

**Authors:** Taylor J. B. Forrester, Olga G. Ovchinnikova, Zhixiong Li, Elena N. Kitova, Jeremy T. Nothof, Akihiko Koizumi, John S. Klassen, Todd L. Lowary, Chris Whitfield, Matthew S. Kimber

**Affiliations:** 1 Department of Molecular and Cellular Biology, University of Guelph, 50 Stone Road E., Guelph, ON N1G 2W1 Canada; 2 Department of Chemistry, University of Alberta, 11227 Saskatchewan Drive, Edmonton, AB T6G 2G2 Canada; 3 Institute of Biological Chemistry, Academia Sinica, Academia Road, Section 2, #128, Nangang, Taipei, 11529 Taiwan; 4 Institute of Biochemical Sciences, National Taiwan University, Section 4, #1, Roosevelt Road, Taipei, 10617 Taiwan

**Keywords:** Glycobiology, X-ray crystallography, Enzyme mechanisms

## Abstract

WbbB, a lipopolysaccharide O-antigen synthesis enzyme from *Raoultella terrigena*, contains an N-terminal glycosyltransferase domain with a highly modified architecture that adds a terminal β-Kdo (3-deoxy-d-*manno*-oct-2-ulosonic acid) residue to the O-antigen saccharide, with retention of stereochemistry. We show, using mass spectrometry, that WbbB forms a covalent adduct between the catalytic nucleophile, Asp232, and Kdo. We also determine X-ray structures for the CMP-β-Kdo donor complex, for Kdo-adducts with D232N and D232C WbbB variants, for a synthetic disaccharide acceptor complex, and for a ternary complex with both a Kdo-adduct and the acceptor. Together, these structures show that the enzyme-linked Asp232-Kdo adduct rotates to reposition the Kdo into a second sub-site, which then transfers Kdo to the acceptor. Retaining glycosyltransferases were thought to use only the front-side S_*N*_i substitution mechanism; here we show that retaining glycosyltransferases can also potentially use double-displacement mechanisms, but incorporating an additional catalytic subsite requires rearrangement of the protein’s architecture.

## Abstract (web summary)

WbbB is a structurally unusual retaining glycosyltransferase. Here, the authors show that WbbB forms an Asp232-Kdo adduct prior to transfer to the saccharide acceptor. Therefore, unlike any previously studied glycosyltransferase, WbbB uses the double-displacement mechanism first proposed in 1953.

## Introduction

Glycosyltransferases (GTs) catalyze the transfer of a saccharide from an activated donor to an acceptor. The leaving group of the donor in this reaction is a phosphate or pyrophosphate ester (generally linked to a mono- or dinucleotide, or a lipid) while the acceptor can be another saccharide, a protein, a lipid, a nucleic acid, or a small molecule. The importance of this class of enzymes is underscored by the fact that GTs comprise 1–2% of encoded proteins^1^. GTs are classified by the Carbohydrate-Active enZYmes (CAZy) database into families based on sequence homology, with 115 families currently recognized^2^. Despite this sequence and functional diversity, all structurally characterized GTs can be classified as having one of five folds, with most being classical Leloir enzymes belonging to either GT-A (with a single modified Rossmann fold domain), or GT-B (with two Rossmann fold domains, separated by a deep catalytic cleft)^1^.

**Fig. 1:**
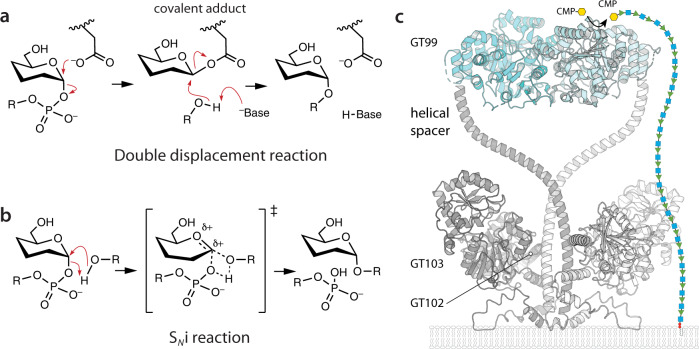
Mechanisms of retaining GTs. **a** Two potential mechanisms have been proposed for retaining GTs. A double-displacement mechanism would follow the pattern generally seen among other enzymes, including glycosyl hydrolases, but has never been unambiguously demonstrated for any GT. **b** Instead, all well-characterized retaining GTs appear to use an S_*N*_i mechanism, where the phosphate dissociates from the donor to form a cationic intermediate, which then reacts with the incoming acceptor. **c** Organization and domain roles of WbbB. The GT99 domain in cyan is based on the experimental structure (5FA0); regions in grey are based on Colabfold^48^ modelling. The undecaprenyl pyrophosphate O-antigen product is shown in symbol form; O-antigen termination occurs only once the O-antigen is long enough to reach the WbbB_GT99_ active site.

GTs can also be broadly classified by whether the configuration of the donor anomeric carbon is inverted or retained during transfer. Inverting GTs are mechanistically well understood. They exploit a single displacement mechanism involving attack on the anomeric carbon of the donor by a nucleophile (generally a hydroxyl group) and a leaving group departing from the opposite face of the ring. In contrast, the mechanism of retaining GTs has been more controversial. Originally, retaining GTs were assumed to proceed via a Koshland double-displacement mechanism, where nucleophilic attack on the donor by an enzyme group produces a covalent enzyme intermediate with inverted anomeric configuration; this adduct then undergoes a second nucleophilic attack by the acceptor, restoring the original anomeric configuration^3^ (Fig. 1a). Retaining glycoside hydrolases (GHs) offer a clear precedent for this mechanism, with one acidic residue in the active site acting as the nucleophile, and a second acidic residue, approximately 5 Å away, activating a water molecule that hydrolyses the covalent intermediate^4,5^. To date there have been two reports suggesting the formation of enzyme-linked adducts in retaining glycosyltransferases. Lairson et al. observed the modification of an Asp residue adjacent to the active site in lipopolysaccharyl-(α−1→4)-galactosyltransferase C (GT-8) when another candidate residue was mutated^6^. In another study, Soya et al. observed that the variants of human α-(1→3)-N-acetylgalactosaminyl-transferase (GT-6) could be glycosylated by a cognate donor when an active site glutamate was replaced with cysteine^7^. Quantum mechanics/molecular mechanics (QM/MM) metadynamics analysis of a bovine GT-6’s mechanism predicts that, while the substrates are organized very similarly to other retaining GTs, the donor saccharide forms an intermediate glutamate-galactose covalent adduct prior to transfer to the acceptor^8^; however, in later experiments on human GT-6, mutation of Glu303 to cysteine or aspartate was found to only slightly slow the reaction^9^. Most reported structures of retaining GTs show no suitable candidate residue positioned to act as the nucleophile, and studies using methods that trap GH intermediates have failed to find covalent adducts^10^, indicating that double-displacement is not the universal retaining GT mechanism. Instead, all current well-studied retaining GTs (including the above two examples) are now believed to use an “internal return” S_*N*_i–like substitution mechanism, where nucleophile approach and leaving group departure occur on the same face of the carbohydrate ring (Fig. 1b)^1,11^. Important evidence for this mechanism comes from detailed QM/MM studies which in multiple retaining GT families – including GT8 (xyloside α1–3 xylosyltransferase and LgcT)^12,13^, GT15 (α−1,2-mannosyltransferase^14^, GT20 (trehalose-6-phosphate synthase),^15^, GT27 (polypeptide UDP-GalNAc transferase 2)^16^, GT44 (Rho glycosylating TcdB toxin)^17^ and GT78 (mannosylglycerate synthase)^18^ - support a dissociative S_*N*_i-like mechanism with a short-lived oxacarbenium ion intermediate that then reacts with the acceptor^19^. Additional evidence for the S_*N*_i mechanism comes from structures showing an appropriate organization of the Michaelis complex, kinetic isotope effects (in trehalose-6-phosphate synthase and TcdB), and the absence of strong evidence supporting competing mechanisms^1,11,17,20^.

3-Deoxy-d-*manno*-oct-2-ulosonic acid (Kdo) is an eight-carbon monosaccharide structurally related to sialic acid. It is an integral part of the lipopolysaccharide (LPS) core, where it links the lipid A moiety to the core oligosaccharide, making it essential in most Gram-negative bacteria. In *Raoultella terrigena* ATCC 33257 (and *Klebsiella pneumoniae* O12) lipopolysaccharide (LPS) biosynthesis, the repeat-unit region of the O-antigen polysaccharide is built by WbbB, a tri-functional membrane-anchored GT. WbbB’s two C-terminal GT domains produce a polymer with a [4)-α-Rha*p*-(1→3)-β-Glc*p*NAc-(1→] repeat unit, while the N-terminal domain of the protein contains a GT99 retaining β-Kdo transferase, referred to here as WbbB_GT99_ (Fig. 1c)^21,22^. The N-terminal transferase domain is separated from the polymerizing GTs by a helical spacer, and adds a single β-Kdo residue to the C-3 hydroxyl group of the terminal rhamnose once the polysaccharide is long enough to reach its active site^21^. Kdo addition blocks further polymerization while creating the epitope recognized by the carbohydrate-binding module of the cognate ABC transporter, allowing export prior to ligation to lipid A-core in the periplasm^23^.

WbbB_GT99_ has an unusual, highly modified GT-B structure; a greatly reduced N-terminal Rossmann fold domain is displaced from, and linked to, the C-terminal domain by a helical insertion domain^21^. This unusual architecture has only so far been seen in GT107, the second known family of retaining β-Kdo transferases, exemplified by the group 2 capsule linker synthesis enzyme KpsC. Interestingly, GT107 also shares multiple key active site residues with GT99, suggesting possible mechanistic similarities^24,25^. Three key features of the WbbB_GT99_ active site are consistent with a possible double-displacement mechanism: (1) Asp232 is a strong candidate for a catalytic nucleophile. This residue is positioned adjacent to the nucleotide phosphate, and absolutely conserved across all known retaining Kdo transferases. A D232A variant in WbbB_GT99_ (and the analogous D160A variant in KpsC) has no detectable activity^21,25^; (2) Glu158 is a candidate for an acceptor activating base in the second reaction step. Glu158 is absolutely conserved across all known retaining Kdo transferases, the E158A enzyme variant is wholly inactive, and the spacing between Asp232 and Glu158 is similar to the spacing between active site acid residues in GH enzymes; 3) His265 is absolutely conserved, and structurally homologous to the ‘HP’ motif histidine in sialyltransferases where this residue protonates the phosphate leaving group during an inverting reaction^26,27^. Acceptor protonation of the phosphate would make this residue redundant in an S_*N*_i mechanism, but an H265A variant is severely catalytically compromised^21^.

In this work, we show that WbbB_GT99_ forms Kdo adducts to Asp232. We also describe the structures of key WbbB_GT99_ reaction intermediates, showing that Asp232 is poised to attack CMP-β-Kdo in the donor complex, that the covalent adduct undergoes a significant rearrangement, and that the ternary complex formed by the rearranged adduct has near-ideal geometry for transferring Kdo from the enzyme to the acceptor. Together, this constitutes strong evidence that WbbB_GT99_ employs a double-displacement mechanism.

## Results

### WbbB_GT99_ forms a covalent adduct between Kdo and Asp232

Details of the kinetic behaviour of an enzyme can give invaluable insight into its mechanism. However, WbbB_GT99_ is exceedingly challenging to characterize kinetically. The donor, CMP-β-Kdo is unstable with a ~30 minute half-life^28^, and must therefore be generated in situ. However, CMP-β-Kdo synthase, the CMP-β-Kdo synthetic enzyme, can only use the minor (~2%) non-lactonized β-pyranose Kdo isomer as a substrate, and donor accumulation is then limited by slow mutarotation^29^ in competition with hydrolysis. These issues make control or even estimation of the donor concentration in an assay very challenging. We attempted to analyze Michaelis–Menten kinetics with respect to the concentration of a synthetic disaccharide acceptor **1** (Supplementary Fig. 1a) using an assay that monitors product formation by HPLC (Supplementary Fig. 1b), but were unable to saturate the enzyme with the available substrate concentration (1 mM). While these data do imply an apparent *k*_cat_/*K*_M_ of 1.00 ± 0.02 × 10^3^ M^−1^ s^−1^ for this substrate, gaining mechanistic insight from WbbB_GT99_ kinetics with available conventional approaches is impractical.

**Fig. 2:**
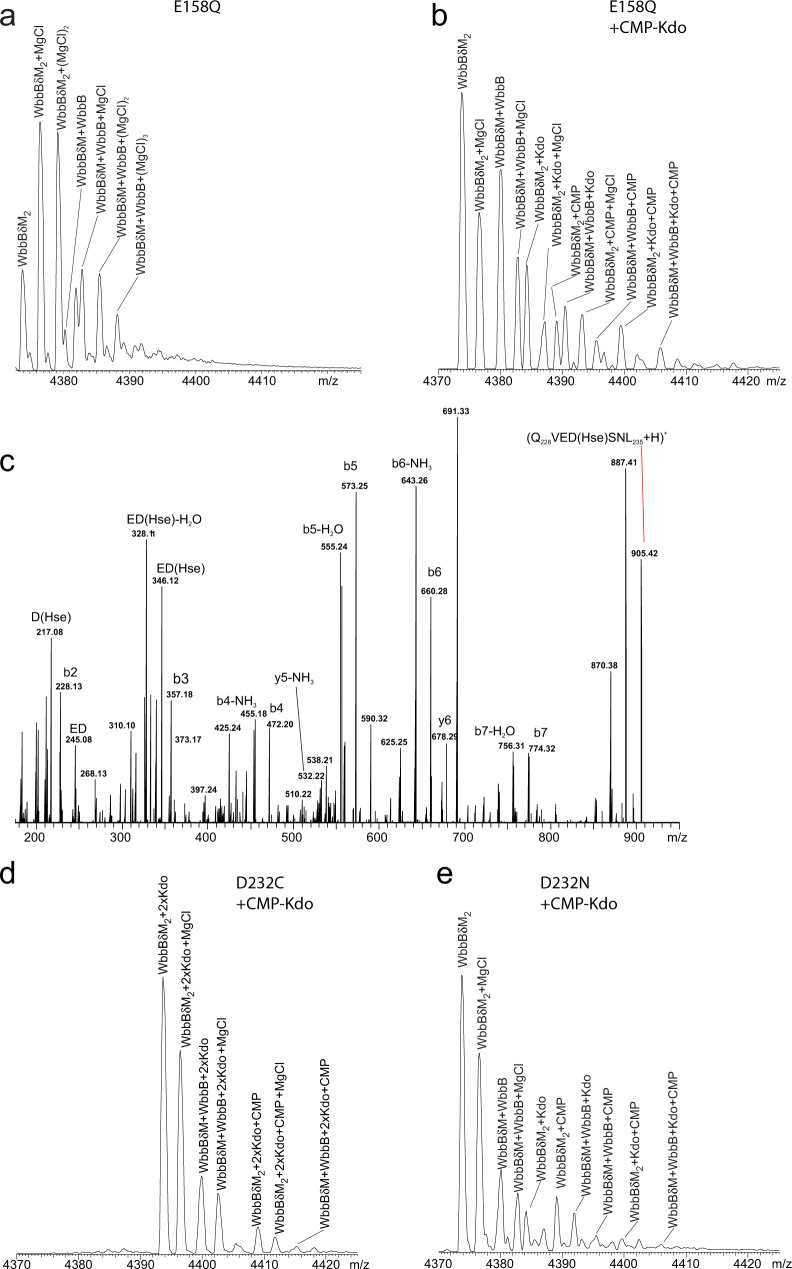
Mass spectrometry evidence for the formation of a WbbB_GT99_ D232-Kdo adduct in different WbbB_GT99_ active site variants. All mass spectra were acquired in positive mode with a UHMR Orbitrap mass spectrometer, and all except **c** were collected for aqueous ammonium acetate solutions (200 mM, pH 7, and 25 °C) and represent the +21 charge state of the dimer of WbbB_GT99_ variants. The N-terminal methionine is removed from WbbB_GT99_ with variable efficiency during expression, giving three major peaks for the unlabeled protein dimer. The addition of a single Kdo residue is calculated to add 220.06 Da to the protein’s mass. **a** Mass spectrum of WbbB_GT99_-E158Q variant purified from ClearColi^TM^. **b** Mass spectrum of the WbbB_GT99_-E158Q variant after reaction with CMP-Kdo reaction mix. Note that for peaks where both CMP and Kdo are present, this method cannot resolve whether Kdo is covalently attached to CMP or WbbB. Source data are provided as a Source Data file. **c** MS/MS spectrum of the singly charged peptide (Q_228_VED(Hse)SNL_235_) precursor ion (m/z 905.42) acquired with a Q Exactive Orbitrap mass spectrometer at a collision energy (CE) of 40 V. The homoserine (Hse) in this peptide was generated by reducing the CMP-Kdo reacted wild-type WbbB with sodium borohydride, which will only reduce carboxylate residues which have been glycosylated. **d** Mass spectrum of WbbB_GT99_-D232C variant after reaction with CMP-Kdo reaction mix. **e** Mass spectrum of WbbB_GT99_-D232N variant after reaction with CMP-Kdo reaction mix.

An important hallmark of a double-displacement GT reaction is the formation of a covalent adduct between the donor saccharide and a nucleophilic amino acid residue in the GT active site. We incubated the wild-type WbbB_GT99_ protein with a CMP-β-Kdo reaction mix containing CTP, Kdo, and *Escherichia coli* KdsB (which generates CMP-β-Kdo in situ), and, after separating the protein from the reaction mix, characterized the product using native mass spectrometry (MS). However, no appreciable Kdo modification was detectable in the resulting mass spectrum (Supplementary Fig. 2a, b). We hypothesized that, in the absence of acceptor, WbbB_GT99_ may use water to attack the adduct, resulting in a hydrolysis reaction that is analogous to the second half-reaction of glycosyl hydrolases^5^. We therefore generated a E158Q variant, which should inactivate the proposed catalytic base, but preserve any important hydrogen bonding interactions. We tested the ability of this variant to turn over in an activity assay where the enzyme was incubated with the CMP-β-Kdo generating mix and acceptor **1**, and then used SDS PAGE to resolve the Kdo-modified fluorescein labelled product from acceptor (Table 1, Supplementary Fig. 3). Like the WbbB_GT99_-E158A variant we previously characterized^21^, this variant is wholly inactive (Table 1). WbbB_GT99_-E158Q (purified from ClearColi^TM^ *E. coli*^30^) appears in these mass spectra as dimers in three distinct forms due to partial removal of the N-terminal methionine during expression (Fig. 2a). After incubation with the CMP-Kdo reaction mix, approximately 13% of each protein peak is converted to a new, 220 Da heavier, peak (Fig. 2b). Because the transfer of a single Kdo residue from CMP-Kdo would add 220.06 Da, we concluded that these peaks correspond to WbbB_GT99_-E158Q modified by a single Kdo residue. Protein expressed in ClearColi^TM^ was used because expression in BL21(DE3) resulted in roughly 20% of this variant being already modified by Kdo upon purification (Supplementary Fig. 2c). ClearColi^TM^ has both known arabinose-5-phosphate isomerases (*kdsD* and *gutQ*) knocked out, and is therefore severely Kdo-depleted, abrogating this in vivo labelling. To identify the site of modification, Kdo labelled WbbB_GT99_-E158Q was digested using pepsin, and characterized using nanoESI (Supplementary Fig. 2d). The Kdo residue was localized to the fragment Q_228_VEDDSNL_235_, consistent with Asp232 (underlined) being the adduct-forming residue. Collision-induced decay of this peptide resulted in neutral loss of Kdo from this fragment (Supplementary Fig. 2e); these experiments confirm the identity of the modified peptide, but do not permit isolation of the Kdo-modified residue.

**Table 1:** Enzymatic activity of WbbB_GT99_ active site variants

WbbB_GT99_ variant	Relative turnover
wt	1^a^
R12A	0.029 ± 0.0054^c^
W20A	0.032 ± 0.0042^b^
W54A	0.012 ± 0.0017^c^
E158Q	N.D.^d^
R163A	0.089 ± 0.0022^b^
D232C	N.D.^d^
D232N	0.0038 ± 0.0021^c^

^a^Variant evaluated at 0.5 μg/ml, with four replicates.

^b^Variants evaluated at 5 μg/ml, with four replicates.

^c^Variants evaluated at 50 μg/ml, with five replicates.

^d^N.D.—not detected; assays results were indistinguishable from a no enzyme control (0.003 ± 0.003) at 50 μg/ml.

To identify the exact site of Kdo modification, we took advantage of sodium borohydride’s ability to reduce glycosylated aspartate residues to homoserine^31^. After incubating wildtype WbbB_GT99_ with the CMP-β-Kdo reaction mix and then adding sodium borohydride, most of the protein (86 ± 7%) was converted to a series of new peaks 14 Da lighter per chain than the unmodified enzyme (Supplementary Fig. 4 and Supplementary Table 1). This mass loss corresponds to that expected for the conversion of a single aspartate to homoserine. Upon pepsin digest, a new 905.42 Da peak was identified, which corresponds to the peptide (Q_228_VED(Hse)SNL_235_), where Hse is homoserine (Supplementary Fig. 5a, b; Supplementary Table 2). Analysis of this peptide by MS/MS confirmed that Asp232 is the sole residue that has been converted to homoserine, and is therefore the only site of Kdo modification within WbbB_GT99_ (Fig. 2c). Interestingly, an 1125.48 Da (905.42 Da + 220.06 Da) peak was also identified within the same MS/MS spectra (Supplementary Fig. 5); this suggests that Hse232 is capable of acting as a nucleophile and forming a Kdo adduct, though the lability of the Kdo adduct again prevents us from confirming the modification site directly via MS/MS experiments.

Both analysis of the CMP co-structure and the above experiments strongly implicate Asp232 as the catalytic nucleophile; we, therefore, sought to identify Asp232 variants that could potentially form stable adducts. Although cysteine is an imperfect structural mimic of aspartate, it is an excellent nucleophile^32^ and can potentially form very stable adducts (as suggested by the recalcitrance of thioglycosides to hydrolysis by neuraminidases^33^). We generated a WbbB_GT99_-D232C variant; this protein was completely labelled when expressed in BL21(DE3), but even protein expressed in ClearColi^TM^ exhibited ~35% labelling (Supplementary Fig. 6a, b) suggesting that additional unidentified enzyme(s) with appreciable arabinose-5-phosphate isomerase activity remains active in this strain. WbbB_GT99_-D232C purified from ClearColi^TM^ could be completely labelled by the addition of the CMP-β-Kdo reaction mix (Fig. 2d). No detectable product was found in the activity assay, implying that Cys232 cannot transfer Kdo to the acceptor (Table 1).

Finally, asparagine is a close structural mimic of aspartate, but both a worse nucleophile and a worse leaving group. The WbbB_GT99_-D232N variant was ~27% Kdo labelled in BL21(DE3), but Kdo-free when expressed in ClearColi^TM^ (Supplementary Fig. 6c, d). Incubating this protein with a CMP-β-Kdo-generating reaction mix again resulted in protein that was Kdo modified, albeit at a lower efficiency than E158Q (~8%) (Fig. 2e). The D232N variant retains ~0.4% of wild type activity, arguing that the WbbB_GT99_-D232N-adduct remains reactive, albeit only capable of slow transfer of Kdo to the protein (Table 1). Together, these findings suggest that Asp232 can form an adduct to Kdo, but significant accumulation of the adduct requires that either the general base is neutralized, or Asp232 substituted with a nucleophile that is a worse leaving group.

### CMP-β-Kdo binding buries the Kdo anomeric carbon adjacent to Asp232

**Fig. 3:**
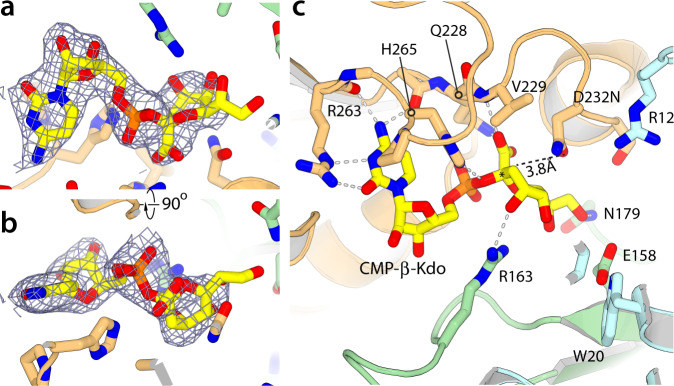
Structure of the WbbB_GT99_ CMP-β-Kdo complex. **a**, **b** Orthogonal views of the electron density for CMP-β-Kdo (blue mesh is the 2mFo-DFc map contoured at 1.0 σ). **c** Details of interactions mediated by CMP-β-Kdo in the WbbB_GT99_ active site. In all structure figures unless noted otherwise, ligands are shown with yellow carbon atoms, C-terminal domain residues in pale orange, N-terminal domain residues in cyan, and regions from the linker/helical domain in green.

Because WbbB_GT99_ appears to use an unreported mechanism, we sought to capture a series of snapshots of the reaction cycle using X-ray crystallography. Capturing WbbB_GT99_ in complex with the CMP-β-Kdo donor is complicated by its instability^28^. To overcome this problem, WbbB_GT99_-CMP complex crystals were grown and then soaked in buffer supplemented with a CMP-β-Kdo generating reaction mix, followed by rapid freezing. We determined structures of WbbB_GT99_ variants with substitutions of Asp232 aimed at slowing, or preventing, adduct formation and subsequent hydrolysis. Of these, the D232A and D232G crystals showed additional density in the site adjacent to the CMP phosphate, but it was too weak to model confidently. We hypothesized that additional contacts from the side chain are required to properly stabilize Kdo in its binding site. However, WbbB_GT99_-D232N crystals soaked with the CMP-β-Kdo synthesis cocktail typically react to form an adduct within the two-minute time frame required for crystal manipulations (see below). Finding conditions that slowed adduct formation without accelerating internal hydrolysis or destabilizing the crystal proved challenging, but crystals grown in an ammonium sulfate condition at pH 7.5 and soaked for 30 min showed appreciable additional electron density in this site (Fig. 3a, b; omit maps are shown in Supplementary Fig. 7a). The density corresponding to the Kdo group is significantly weaker than that for the nucleotide but refinement of the structure using both CMP and CMP-β-Kdo results in 25% occupancy of CMP-β-Kdo in the active site, with atomic displacement parameters (ADPs; B-factors) similar to surrounding residues. Because of the partial Kdo occupancy (complicated by the presence of competing water molecules), the O5, O7, and O8 hydroxyl groups lack strong peaks in the density map (though density is apparent at 0.5 σ). This weak binding implies that the Kdo ring is not tightly bound, with binding predominantly driven by interactions with the nucleotide and the carboxylate group. Despite the uncertainty in the fine details, the overall binding of the substrate is clear and unambiguous.

Available protein structures containing CMP-β-Kdo (as a product in CMP-β-Kdo synthase, 1GQC; and as an inhibitor in arabinose-5-phosphate isomerase, 3ETN)) both show β-Kdo in the chair conformation, which is generally the lowest energy conformation for pyranose rings^34^. In WbbB_GT99_, the CMP-β-Kdo density is not consistent with this conformation, with Kdo instead refining to the higher-energy skew-boat (^O^*S*_*3*_) conformation. However, limitations in the quality of the map preclude complete confidence in this assignment (Fig. 3c and Supplementary Fig. 8a). The C1 carboxylate group forms hydrogen bonds with both the backbone and side chain amide of the invariant Gln228 from the QxxxD motif. In addition, O4 forms hydrogen bonds with Arg163, as well as the phosphate-linking oxygen, while O5 and O7 form a hydrogen bond but are otherwise facing the solvent channel. The exocyclic O8 hydroxyl group hydrogen bonds to both Glu158 and Asn179. In this conformation, Asn232(=Asp) packs against the edge of the carboxylate and the exposed ring of Kdo, but interacts only with aliphatic groups and makes no hydrogen bonds. Notably, both polar atoms are within van der Waals contact distance of the anomeric carbon (the closer is 3.8 Å away) and can be brought closer (to within 2.8 Å) by rotation around the Cβ–Cγ bond. In addition to Asp232, the vicinity of the anomeric carbon is crowded by Val229, His265, Arg163 and the axial hydroxyl group at C4. The anomeric carbon is therefore completely buried in this conformation, precluding (absent a major reorganization of the active site) the acceptor approaching this atom as required for an S_*N*_i reaction. We previously showed that the Q228A variant is severely catalytically compromised, and H265A also has reduced activity^21^, consistent with the relevance of this binding mode.

### WbbB_GT99_ repositions α-Kdo adducts into a distinct sub-site

**Fig. 4:**
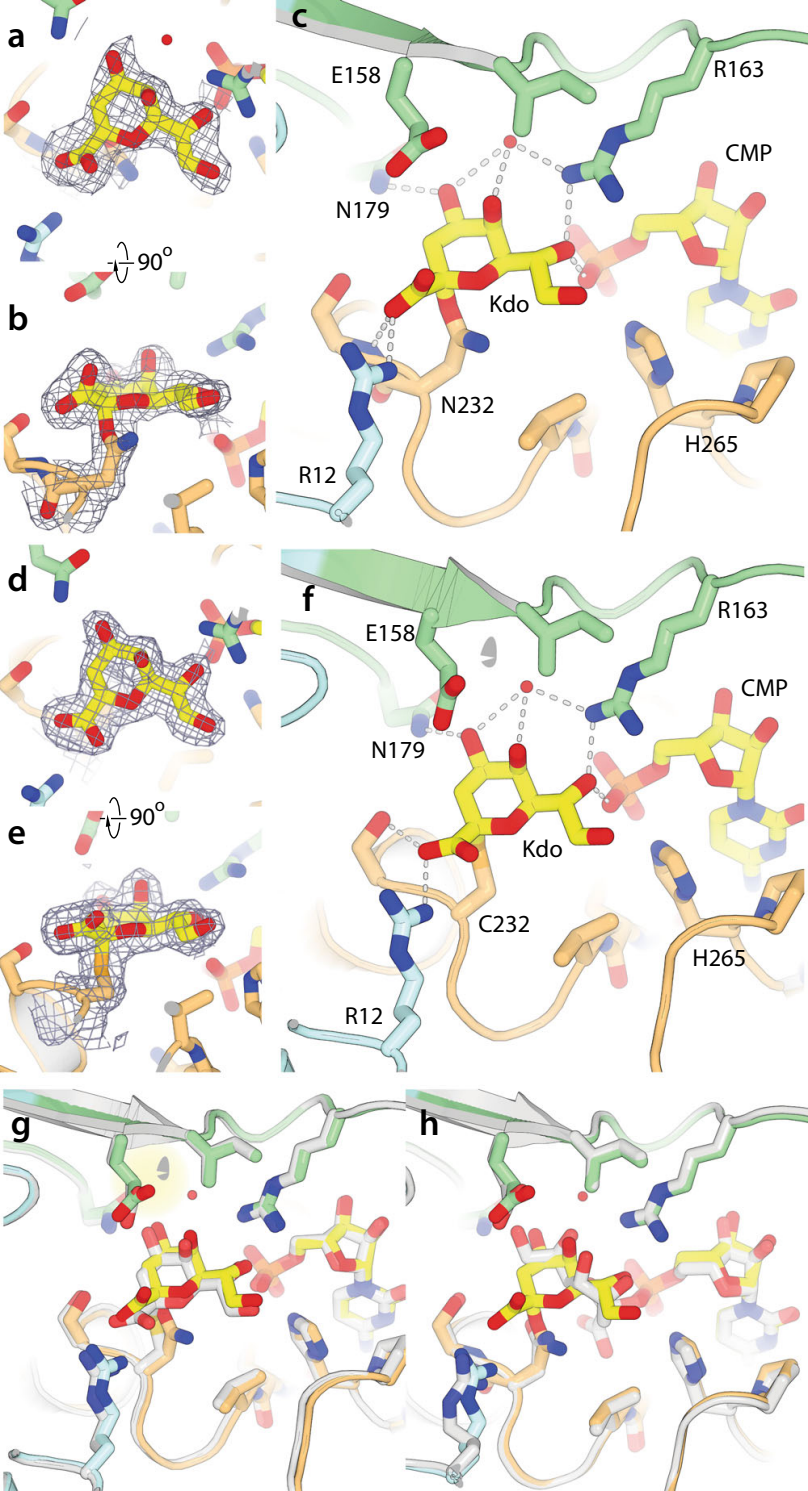
Structures of α-Kdo adducts. **a**, **b** Electron density for the WbbB_GT99_-Asn232–α-Kdo adduct (2mFo-DFc map contoured at 1.0 σ). **c** Details of the interactions made by the WbbB_GT99_-Asn232–α-Kdo covalent intermediate adduct in the active site. **d**, **e** Electron density for the WbbB_GT99_-Cys232–α-Kdo adduct, (2mFo-DFc map contoured at 1.0 σ). **f** Details of the interactions made by the WbbB_GT99_-Cys232–α-Kdo covalent intermediate adduct in the active site. **g** Superposition of the WbbB_GT99_-Asn–α-Kdo and WbbB_GT99_-Cys–α-Kdo adduct structures. The WbbB_GT99_-Cys–α-Kdo adduct structure is shown in white. **h** Superposition of the CMP-β-Kdo complex (in white) on the WbbB_GT99_-Asn232–α-Kdo adduct structure. The Kdo residue is completely flipped in the active site, but the ridge of hydroxyl groups O4–O8 mediate interactions with the same residues in both orientations.

We next determined the structure of both the WbbB_GT99_-D232N (at 1.9 Å) and WbbB_GT99_-D232C (at 1.95 Å) variants as Kdo adducts. The WbbB_GT99_-D232N variant crystal was grown as a CMP complex, then soaked with a CMP-β-Kdo-generating reaction mix for five minutes before freezing. For both variants, clear additional density was present that could readily be modelled as an α-Kdo with an unambiguous linkage to the anomeric carbon. After refinement, in the WbbB_GT99_-Asn232–Kdo structure, Kdo has a 0.7 occupancy, with ADPs similar to those of surrounding side chains (suggesting around 70% conversion to the adduct), while the resulting electron density clearly defines the positions of all atoms (Fig. 4a, b and Supplementary Fig. 7b). Significantly more adduct is apparent in the structure than was observed by LC/MS, supporting the idea that this adduct forms quickly, and then undergoes gradual hydrolysis with a half-life on an hour-time scale. The WbbB_GT99_-D232C-Kdo adduct was generated by crystallizing a protein that had been spontaneously modified by the CMP-β-Kdo present during expression in *E. coli* BL21(DE3). In this structure, Kdo is at full occupancy, has ADPs similar to those of surrounding residues, and electron density quality comparable to that of fully buried amino acids (Fig. 4e, f and Supplementary Fig. 7c). The Asn- and Cys- Kdo adduct structures refine with α-Kdo in a very similar position, with only small shifts (0.29 Å r.m.s.d. for Kdo compared to 0.24 Å for the protein), with the largest shifts in the anomeric carbon (0.6 Å) and carboxylate residue (Fig. 4c, g and Supplementary Fig. 8b, c). In the WbbB_GT99_-Asn232–α-Kdo structure, Kdo is attached to the syn lone pair of the Asn, consistent with the organization of the CMP-β-Kdo complex structure (Fig. 4c) and modification site in retaining GHs^5,35^. The map does not unambiguously resolve whether the Nδ or Oδ of Asn232 is linked to Kdo, but WbbB lacks a base analogous to that used by N-glycosyltransferases to deprotonate the amide nitrogen nucleophile^36^ and the WbbB_GT99_-D232N adducts remain susceptible to both hydrolysis and transfer, suggesting that they are not as stable as N-linked glycans. A clear analogy can be found in retaining hexosaminidases, where the acetamide oxygen acts as a nucleophile, attacking the anomeric carbon to form a cyclic oxazolinium ion intermediate that is stabilized by adjacent acidic groups, and subsequently cleaved by enzyme catalyzed hydrolysis^37^. In WbbB, which lacks nearby stabilizing acidic groups, the resulting linear carboximidate ion likely N-deprotonates to form a long-lived intermediate.

The most striking feature of the adduct structure is the dramatic reorganization of the Kdo group relative to the CMP-β-Kdo complex, with the C1 carboxylate moved furthest away from the leaving group phosphate, the exocyclic O8 group closest, and the anomeric carbon shifted 6.1 Å from the leaving group phosphate oxygen. The Kdo adduct is stabilized in the active site by multiple contacts with the protein. The carboxylate group interacts with Arg12, as well as Ser233; in the WbbB_GT99_-D232N–Kdo structure, the hydrogen bond is to Ser233 N, while a ~30° rotation of the carboxylate group reorients this oxygen in the WbbB_GT99_-D232C–Kdo structure to instead hydrogen bond with the Ser233 Oγ. The Kdo O4 forms hydrogen bonds with Glu158 and Asn179 (as well as a structured water); O6 forms a pair of hydrogen bonds with Arg163 as well as the CMP phosphate oxygen. The latter interaction is of particular note, as it suggests that CMP remains an integral part of the Kdo-covalent adduct binding site, and likely remains bound through the reaction cycle. However, the repositioning of the substrate means that the anomeric carbon is now distant from the phosphate. Thus, in the case of WbbB_GT99_ at the very least, the nucleotide phosphate is unlikely to act as a general base in the second half reaction, as had been hypothesized^1^.

Comparison of the CMP-β-Kdo structure to the Kdo adduct structure suggests that structural self-similarity within the Kdo residue is exploited by the enzyme to allow two distinct binding modes. In particular, O4 and O8 in CMP-β-Kdo each make a pair of hydrogen bonds with the protein. In the α-Kdo adduct structure, the sugar is positioned so that O7 and O4, respectively, sit in almost identical positions and exploit the same hydrogen bonding interactions (Fig. 4h).

### The acceptor binds predominantly to the N-terminal domain

**Fig. 5:**
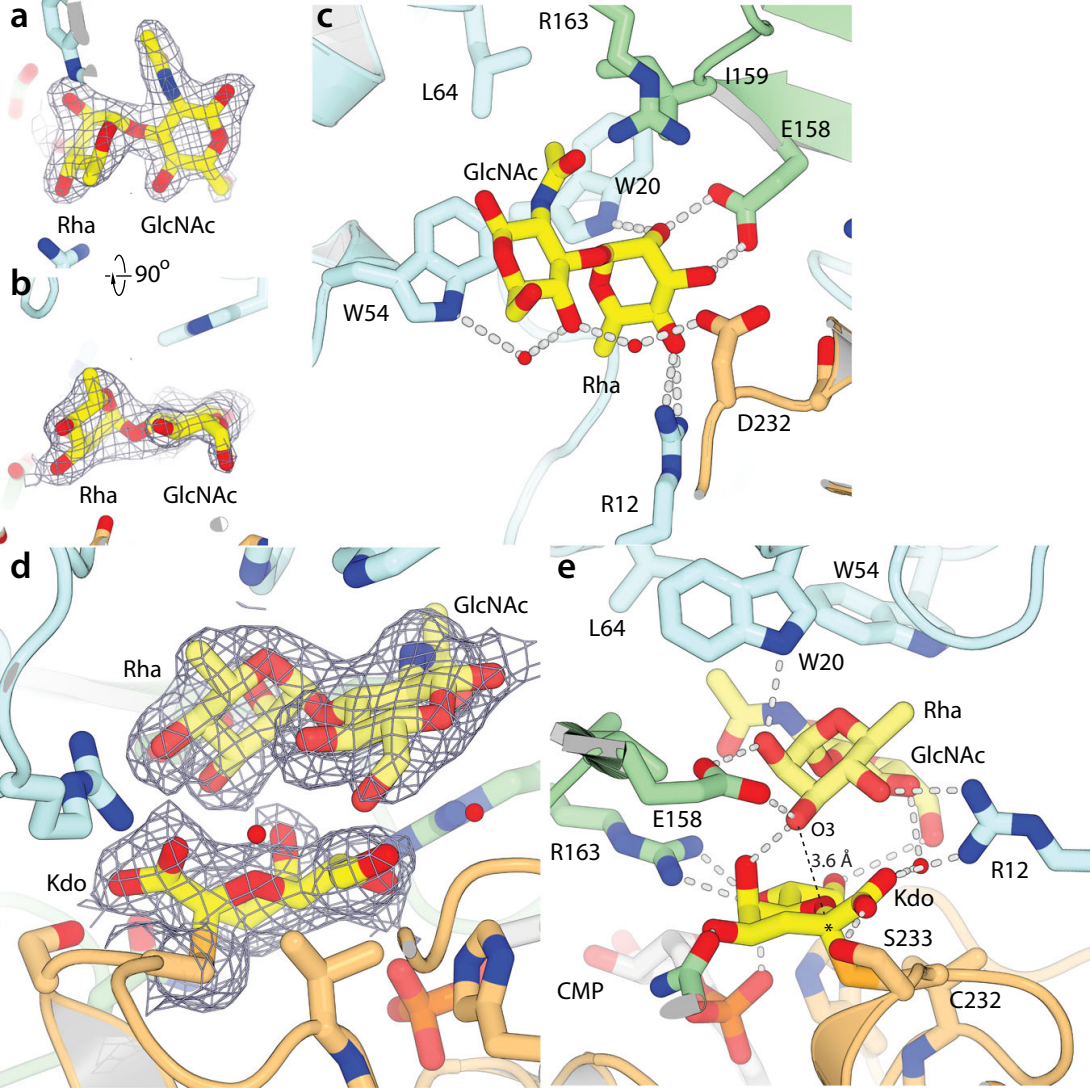
Structures of the acceptor and ternary complexes. **a**, **b** Orthogonal views of the electron density (2mFo-DFc map contoured at 1.0 σ) for the acceptor bound in the WbbB_GT99_ active site. **c** Details of the interactions mediated by the acceptor in the active site. **d** Electron density for the ternary complex, contoured around Cys232, Kdo, and the acceptor (2mFo-DFc map contoured at 1.0 σ). **e** Details of the ternary complex structure. Neither the donor nor acceptor shifts appreciably from their respective binary complexes, and the donor and acceptor interact extensively. This complex places the O3 hydroxyl group of rhamnose within 3.6 Å of the anomeric carbon of Kdo (marked with an asterisk) and in line with the Kdo(C2)–C232Sγ bond.

We determined the structure (at 2.3 Å resolution) of the wild-type enzyme co-crystallized with 5 mM of both CMP and a disaccharide acceptor (α-Rha-(1→3)-β-GlcNAc-C8-methoxybenzamide, **2**) (Supplementary Fig. 1a). Disaccharide density is clearly visible in both active sites, and after building refines with good occupancy (0.9/0.8 and 0.8/0.7 for Rha/GlcNAc in the A and B chains, respectively). The ADP values are similar to those of both the surrounding amino acids and to the Wilson B-factor, indicating that the ligand is well-ordered within its binding site. Density for the rhamnose is slightly better defined, but all heavy atoms are clear for both residues (Fig. 5a, b and Supplementary Fig. 7c). The acceptor binds to the opposite side of the pocket from Kdo, making interactions exclusively with residues from the N-terminal α/β domain (Fig. 5c and Supplementary Fig. 8c). Glu158 was previously identified as a residue being absolutely required for the reaction^21^, and makes a pair of close hydrogen bonds (both 2.6 Å) to the O3 and O4 hydroxyl groups of rhamnose. Other important hydrogen bonding interactions include O2 to the indole nitrogen of Trp20 and O4 to Arg12, as well as to a structural water molecule. The C6 methyl is in van der Waals contact with Arg12 and Pro17. The GlcNAc residue is stacked on Trp54, with the methyl of the acetyl group stacking on Trp20. The amide nitrogen of the GlcNAc points to the indole ring of Trp54, making a strong interaction with the π electrons^38^. Both Trp20 and Trp54 are important for turnover, with Trp54Ala having a larger effect (Table 1), consistent with its extensive van der Waals contacts with the GlcNAc acceptor residue. The methyl group of the acetyl moiety sits in a well-defined non-polar pocket, with van der Waals contacts with Trp20, Trp54, Leu64, and Ile159. Interestingly, none of the hydroxyl groups of the GlcNAc residue form hydrogen bonds with the protein, although some do interact with well-ordered water molecules.

### Ternary complex structure

Finally, we determined the structure of a ternary complex of WbbB_GT99_ at 2.4 Å; this structure has the Cys232–Kdo covalent adduct and acceptor disaccharide **2** bound in the active site. The D232C variant was chosen as it closely mimics the D232N structure, but readily reacts with CMP-Kdo to completely label the protein; the adduct neither hydrolyzes nor reacts with the acceptor substrate. The density for the Kdo adduct and the disaccharide acceptor is clear in the resulting map, with all heavy atoms in well-defined density at 1.0 σ (Fig. 5d and Supplementary Fig. 7e); all monosaccharides have occupancies > 0.9, and ADP values similar to those of surrounding amino acids. Although these crystals take approximately one month to form, there is no indication of any product in the map. While the anomeric carbon in the D232C adduct is positioned 0.7 Å closer to Cα than in the D232N adduct (as the missing methylene carbon is partially compensated for by the longer C–S bonds), neither the protein, the Kdo adduct, nor the acceptor in the ternary complex move significantly relative to their positions in the binary complex. This suggests that any structural adjustments necessary to accommodate the slightly shorter side chain of cysteine are minor.

Within this ternary complex, all protein–Kdo and protein–acceptor interactions observed in their respective binary complexes are maintained. In addition to these interactions, the Kdo and acceptor saccharides also interact extensively, with the Rha–GlcNAc disaccharide stacking closely on the α-Kdo so that their surfaces are in van der Waals contact (Fig. 5e and Supplementary Fig. 8e). In addition, hydrogen bonds form between O5 of Kdo and O3 of Rha, and between O8 of Kdo and O6 of the GlcNAc; a water molecule also bridges the Kdo carboxylate O1 and O4 of the GlcNAc. The Kdo carboxylate O1 and Rha O4 are also within 2.9 Å of each other, but both form hydrogen bonds to Arg12, keeping them in close proximity. The R12A and R163A variants are both catalytically compromised, with R12 being more severely so (Table 1), consistent with a key role in organizing this ternary complex. The saccharides in this ternary complex show a high degree of structural complementarity, with the extensive interactions mediated by the Asp232–α-Kdo adduct potentially helping drive affinity for the acceptor. The ability to bind the acceptor to the donor-free active site may indicate that WbbB_GT99_ does not have obligate donor-first binding; however, the contributions of the enzyme-linked donor adduct to acceptor binding suggests that this species may have a higher affinity for the acceptor than other states, suggesting that the enzyme might show ordered bi-bi kinetics.

In this complex, the O3 of rhamnose (which accepts Kdo) is hydrogen bonded to the carboxylate group of Glu158. The Glu158 variants E158A^21^ and E158Q (Table 1) produce no detectable product, while E158Q allows the accumulation of appreciable Asp232–Kdo adducts, presumably by reducing hydrolysis; together, this strongly suggests that this residue acts as a general base. Rhamnose O3 also accepts a hydrogen bond from the Kdo O4, leaving the remaining electron pair free and pointed in the direction of the Kdo anomeric carbon. Rhamnose O3 is 3.6 Å from the anomeric carbon of Kdo (close enough to be subject to van der Waals repulsion), and directly in line (the Rha O3–Kdo C2–Cys232S_γ_ angle is 172°) for nucleophilic attack. This complex, therefore, is organized with a near-ideal geometry and chemical environment for nucleophilic attack on the anomeric carbon by Rha O3, with Asp232 as the leaving group.

### Proposed mechanism

**Fig. 6:**
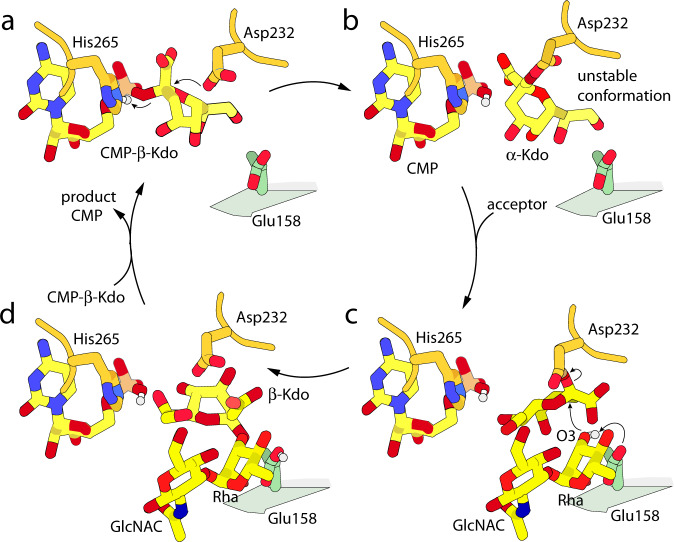
Proposed mechanism for WbbB_GT99_ retaining β-Kdo transferase. **a** CMP-β-Kdo binds in the active site. Asp232 carboxylate performs a nucleophilic attack on the anomeric carbon, while a proton is transferred from His265 to the leaving phosphate group. **b** Immediate product of the first half reaction. This conformation has not been directly observed and is assumed to be unstable and short-lived. **c** Reorganized Asp232–α-Kdo intermediate complex. While the acceptor can bind to the empty active site, the additional interactions mediated by Asp232–α-Kdo imply that this state has significantly higher affinity. The O3 hydroxyl group, activated by the general base Glu158, attacks the anomeric carbon of Kdo in a second inverting reaction, with Asp232 as the leaving group. **d** Product of the second reaction; this complex also appears to be unstable, most likely due to the inversion of the anomeric configuration of Kdo, as well as possible close contacts with Asp232, and has not been directly observed.

The structures described above capture a series of states that suggest a catalytic mechanism. As depicted in Fig. 6 (and Supplementary Fig. 9), CMP-β-Kdo binds adjacent to Asp232 in a strained conformation; Asp232 attacks the anomeric carbon, with CMP acting as the leaving group. This inverting reaction results in the formation of an Asp232–α-Kdo adduct. Relaxing the strained skew-boat pyranose conformation of the Kdo ring into a more relaxed chair moves the C2 anomeric carbon closer to the Oγ atom of Asp232, analogous to the mechanism observed in retaining GHs^5^. This adduct then rearranges in the active site, with CMP remaining bound. The acceptor seems most likely to bind at this point as the additional interactions afforded by the Kdo should help stabilize binding. However, the acceptor would not clash with CMP-β-Kdo, and so could potentially bind earlier. The acceptor is activated via a hydrogen bond between the general base, Glu158, and the O3 hydroxyl group, allowing a second inverting reaction that transfers Kdo to the O3 of rhamnose with a second inversion of configuration, forming the β-Kdo-(2→3)-Rha terminating motif. Sialic acids can react via cationic intermediates that are unusually long-lived^39^; because Kdo resembles sialic acid in having both a 3’ deoxy carbon and an anomeric carbon carboxylate group, these reactions, by analogy, could potentially proceed via an intermediate with considerable dissociative character. The product of Kdo transfer appears unstable in the active site (possibly promoting rapid release) as wild-type crystals soaked in 5 mM product did not show any easily interpretable additional electron density. It should be noted that all observed structures of intermediates show minimal shifts within the active site in response to substrate binding or turnover, with only some minor rotameric shifts in Glu158, Arg12, and Asx232 (Supplementary Fig. 10c), suggesting a high degree of preorganization of the catalytic site.

## Discussion

The evidence presented above strongly argues that WbbB_GT99_ is a retaining glycosyltransferase that uses a double-displacement mechanism analogous to that used by retaining glycoside hydrolases. This mechanism has been previously proposed, but, after further study, every prior candidate has proven to instead use the S_*N*_i mechanism. What was not anticipated from analogies with GH enzymes is the considerable additional complexity apparent in the WbbB_GT99_ double-displacement mechanism. First, WbbB_GT99_ has two distinct donor saccharide-binding modes, with two distinct reaction sites – one to transfer Kdo from CMP-β-Kdo to Asp232, and a second site to transfer Kdo from Asp232–α-Kdo to the acceptor. This contrasts with analogous retaining GHs, where the enzyme–saccharide adduct has an altered ring conformation, but remains bound in the same location in the active site. In GHs, a water molecule occupies a site previously occupied by the leaving group to attack and resolve the enzyme adduct intermediate. We believe that this additional complexity evinced in WbbB_GT99_ is imposed by the conflicting requirements for both stabilizing the nucleotide leaving group and activating the saccharide acceptor. Discussion regarding the organization of the reaction intermediate in double-displacement reactions has typically posited that the nucleotide phosphate remains bound and acts as the general base in the second reaction step^1^. However, if this species remains adjacent to the anomeric carbon of the donor saccharide, it should sterically impede the acceptor from approaching the same atom. Alternatively, the acceptor saccharide and nucleotide could potentially compete for a common binding site, but this requires that the terminal acceptor residue is specifically recognized, and also catalytically activated, by a sub-site that also equally recognizes (and protonates) the phosphate. Repositioning the donor adduct uncouples these two requirements, resulting in two sub-sites that are independently optimized to either form the enzyme adduct, or to transfer Kdo to an acceptor. This problem is presumably less acute for hydrolases (including GHs and serine proteases) because the “acceptor”, water, is small and present at such high concentrations that it saturates even low affinity sites.

The highly unusual mechanism of WbbB_GT99_ (and presumably also KpsC, whose active site contains equivalents of both Asp232 and Glu158^25^) is, in turn, the likely driver behind the highly unusual architecture of these enzymes. The first half-reaction appears to use residues contributed primarily by the C-terminal α/β domain via motifs inherited from an inverting sialyltransferase-like ancestor^21,26,40^. For the second half reaction, all residues (with the exception of Asp232 and Ser233) contacting the Kdo adduct or acceptor are from the N-terminal domain or the inserted α/β domain linker region. To achieve a suitable active site, the N-terminal α/β domain is repositioned and reoriented (relative to its canonical position in GT-B enzymes) by the packing of a helical sub-domain between. This opens up considerable space in the active site, which is then partially filled by an extended β-hairpin motif that packs in this space and provides key catalytic residues (Glu158 and Arg163), while key binding motifs (Arg12, Trp54, Leu64) are provided by extended helices and loops inserted into the canonical GT-B transferase architecture.

In light of the complexity of this WbbB_GT99_ double-displacement mechanism, why did retaining β-Kdo GTs not simply evolve to use the more common S_*N*_i mechanism? One obvious difference between Kdo transfer and the reactions catalyzed by other retaining enzymes is the presence of the carboxylate group on the anomeric carbon; this additional bulk likely makes it more difficult for the acceptor to approach the anomeric carbon productively, with additional crowding from the axial substituent at O4. This idea is consistent with the observation that retaining Kdo transferases (WbbB_GT99_ and KpsC_GT107_) are currently the only known examples of retaining ulosonic acid transferases, while there are seven known families of inverting sialyltransferases (GT29, 38, 42, 52, 80, 97 and 100) and one inverting Kdo transferase (GT30).

In summary, the retaining Kdo transferase WbbB uses a double-displacement reaction rather than the generally observed S_*N*_i-like mechanism. However, this reaction is complicated by an unexpected rearrangement of the covalent adduct that is possibly required to avoid binding competition between the donor leaving group and acceptor. This, however, adds considerable complexity to the catalytic site, and multiple residues in the active site make important contributions to the reaction. In GTs that use an S_*N*_i-like mechanism, in contrast, the protein positions the acceptor appropriately relative to the donor but individual side chains do not participate directly in the reaction^41^. This suggests that double-displacement is likely to evolve far more rarely in GTs than S_*N*_i-like mechanisms. Indeed, if other double-displacement GT families exist in nature, they most likely mediate reactions where optimal S_*N*_i geometry would be similarly frustrated by steric considerations. Our results suggest that researchers seeking to identify further examples of this double-displacement mechanism might use bioinformatics tools to screen for glycosyltransferases where pairs of absolutely conserved acidic residues are positioned with their carboxylate groups spaced approximately 7 Å apart (perhaps using AlphaFold models where experimental structures are unavailable). In addition, borohydride reduction followed by mass spectrometry analysis has promise as a robust and efficient screening tool for confirming candidate enzymes, with the main experimental requirement being access to the cognate donor nucleotide sugar.

## Methods

Reagents were purchased from Fischer Scientific unless specified elsewhere.

### DNA methods

*E. coli* K-12 CMP-Kdo synthetase (KdsB) from was a gift from H. Brade (Research Centre Borstel, Leibnitz Centre for Medicine and Biosciences, Borstel, Germany). The construction of the WbbB_GT99_-His_6_ pET28a expression construct is described in^21^. Protein mutants were generated by site directed mutagenesis using the Quikchange method and primer pairs listed in Supplementary Table 3. Briefly, mutant amplicons were generated using an in-house recombinant stock of *Pfu* X7 DNA polymerase (gifted by Dr. Dinesh Christendat, University of Toronto) according to the Promega *Pfu* DNA polymerase protocol. WbbB_GT99_-His_6_ pET28a plasmid was used as the template after purification using PureLink Quick Plasmid Miniprep Kit (Invitrogen) according to the manufacturer’s instructions. DpnI (New England Biolabs) was then used to digest parental DNA in the PCR samples according to the manufacturer’s instructions. DpnI-treated samples were then transformed into *E. coli* DH5α^42^ chemically competent cells produced in-house using the heat-shock method, plated on LB agar supplemented with 50 µg/mL of kanamycin and incubated overnight at 37 °C. Single isolated colonies were used to inoculate 5 mL of LB media supplemented with 50 µg/mL of kanamycin which were incubated overnight at 37 °C. Variant plasmids were then purified as described above and transformed into *E. coli* BL21 (DE3)^43^ chemically competent cells produced in-house as described above. Glycerol stocks were then prepared for the *E. coli* BL21 (DE3) constructs for future use from saturated LB cultures prepared from a single isolated colony. All constructs were sequenced at the Genomics Facility of the Advanced Analysis Centre at the University of Guelph to confirm only the desired mutations had been obtained.

### Protein expression

WbbB_GT99_-His_6_ and its variants were generally expressed in *E. coli* BL21 (DE3); the E158Q, D232N and D232C variants were expressed in ClearColi^TM^ BL21 (DE3) cells (Lucigen) when unlabelled protein was needed. A scraping from a glycerol stock was used to inoculate 5 mL of LB media supplemented with 50 µg/mL of kanamycin and incubated overnight at 37 °C. All the overnight culture was added to 1 L of 2xYT media also supplemented with 50 µg/mL of kanamycin and grown at 37 °C until an OD_600nm_ of 0.6–0.8 was achieved. IPTG (dioxane-free) was then added to a final concentration of 1 mM, followed by overnight incubation at 16 °C. Cells were harvested at 5000 x *g* for 30 minutes and either used immediately or stored at −70 °C. The cell pellet was resuspended in 35 mL buffer A (50 mM Tris, 250 mM NaCl, pH 8.0) containing 20 mM imidazole and 1 mM phenylmethylsulfonyl fluoride (Acros Organics). The IMAC purification step was performed using an Akta FPLC, with 2 × 1 mL HisTrap Crude FF columns attached in series (GE Lifesciences), and the column eluted with a linear gradient of Buffer A supplemented with 20 – 500 mM imidazole (Acros Organics) over 20 mL. Elutions were analyzed through SDS-PAGE and Coomassie staining. Fractions containing purified protein were desalted into a buffer containing 20 mM Tris-HCl pH 8.0 and 150 mM NaCl (5 mM β-mercaptoethanol for crystallography) for use in downstream experiments using an Akta FPLC and a HiPrep 26/10 desalting column (GE Lifesciences). Protein concentration was checked using A_280_ (extinction coefficient 35,300 M^−1^ cm^−1^ for W20A and W54A, 40,800 M^−1^ cm^−1^ for all others), and the folding of all protein variants was confirmed using the differential-scanning fluorescence method. Proteins were stored at −70 °C following flash freezing in liquid nitrogen.

### Synthesis of acceptors

The synthesis of acceptor **1** was described previously^21^. The synthesis of **2** is described in the Supporting Information (Supplementary Fig. 11).

### Reaction assays

A reaction was prepared containing 100 mM Tris pH 8.0, 10 mM MgCl_2_, 2 mM Kdo, 5 mM CTP, 0.079 mM synthetic acceptor **1**, and 0.5, 5, or 50 μg of a given WbbB_GT99_ variant; the reaction was initiated by the addition of 2 μg of KdsB. Reactions were incubated at 37 °C for 8 min, and then stopped by incubating at 95 °C for 5 min. 2 μL of the reaction mix was then added to loading buffer (without bromophenol blue) and an aliquot was separated on a 15% resolving gel in Tris-glycine buffer. The gel included 0.0125% SDS, the loading dye 0.25% SDS, and the running buffer 0.0125% SDS. Samples were run for 1.75 h at 100 V. Gels were imaged using transillumination with a Chemi-Doc (Bio-Rad) operated with Quantity One v4.6.9 ensuring no pixel saturation; densitometry was performed in ImageJ v152 and relative activities calculated by comparing product generated by a given protein variant against the wild-type enzyme on the same gel. Reported values are based on 4 technical replicates (5  μg enzyme reactions) or 5 technical replicates (50 μg enzyme reactions) that were measured once. Each replicate was performed independently using freshly thawed protein from the same purification preparation.

### Enzyme Kinetics

A 50 µL reaction containing a final concentration of 100 mM Tris-HCl buffer (pH 8.0), 10 mM MgCl_2_, 5 mM CTP, 2.6 mM Kdo, (5–1000 µM) synthetic acceptor **1**, and 4 µg KdsB was incubated at 20 °C for 5 min. WbbB_GT99_ was then added to a final concentration of 543 nM (1.25 µg) and incubation at 20 °C continued. 10 µl aliquots were removed after 2, 5 and 8 minutes and mixed with equal amount of cold acetonitrile to quench. Samples were mixed vigorously, and protein then pelleted at 21,000 x g for 10 min. Samples were analyzed by anion exchange HPLC using an Agilent Technologies 1260 Infinity II system equipped with 1260 Infinity II Variable Wavelength Detector. A DNAPac PA100 column (4 ×  50 mm) was used for chromatographic separation with the following solvents: water (A), acetonitrile (B), and 2 M ammonium acetate (C). The mobile phase gradient was as follows: 20% B was kept constant throughout the run; initial conditions were 0% C; increasing to 45% C in 15 min; hold 45% C for 1 min; decreasing to 0% C in 4 min; 15 min re-equilibration. The flow rate was maintained at 0.5 ml/min, and the column temperature was set to 40 °C. The elution profile was monitored by UV absorbance at 490 nm. Unreacted acceptor and product eluted at 5.4 min and 7.2 min, respectively. Acceptor and product peaks were integrated manually using OpenLAB CDS ChemStation vC.01.07 SR3, and the resulting areas used to provide a ratio percentage of acceptor and product in the reaction. This factor was multiplied by the molarity of the sample to determine the concentration of product at each time point. Progression curves were then plotted; time points between 2 and 8 min showed good linearity and were used to calculate initial velocities (V_0_). Data was plotted and analyzed in GraphPad Prism v8.3. Reported values are based on 3 independent technical replicates that were measured once at each time-point, using freshly thawed protein from the same purification preparation.

### Mass spectrometry sample preparation

WbbB variant stock solutions were exchanged into aqueous 200 mM ammonium acetate (pH 7) using Amicon 0.5 mL microconcentrators (EMD Millipore, Billerica, MA) with a MW cutoff of 10 kDa, and stored at −20 ^o^C until needed. WbbB-_GT99_-Kdo adducts were prepared by incubating a mixture of 5 mM CTP, 5 mM Kdo, 10 mM MgCl_2_, 0.9 mg/ml KdsB and 8 mg/ml WbbB in 200 mM aqueous ammonium acetate (pH 8) at room temperature (22.5 ^o^C) for 3 h. After 3 h, this reaction mixture was buffer exchanged into 200 mM ammonium acetate (pH 7) as described above. For the sodium borohydride reduction experiments, a solution containing 5 mM CTP, 5 mM Kdo and 10 mM MgCl_2_, 0.9 mg/ml KdsB and 8 mg/ml WbbB_GT99_-wildtype were mixed and incubated for 5 min at room temperature. 0.13 mg NaBH_4_ dissolved in 1 μl 200 mM aqueous ammonium acetate (pH 8.5) was then added to a final concentration of 6.5 mg/ml with a total volume of 20 μl, and the sample vortexed and then incubated for a further 3 h at room temperature. Finally the sample was buffer exchanged into 200 mM ammonium acetate (pH 7) as described above. The resulting samples were then analysed using native mass spectrometry (nMS) and/or MS/MS analysis pepsin digests as described below. Six technical replicates of the NaBH_4_ reduction experiments were performed, with similar results.

### Native mass spectrometry

Native MS measurements were performed in positive mode using a Q Exactive Ultra-High Mass Range Orbitrap (UHMR) mass spectrometer (Thermo Fisher Scientific, Bremen, Germany) equipped with a nanoflow ESI (nanoESI) source. NanoESI tips with ~2 μm outer diameters (o.d.) were produced from borosilicate capillaries (1.0 mm o.d., 0.68 mm inner diameter) using a P-1000 micropipette puller (Sutter Instruments, Novato, CA). The nanoESI spray voltage of ~0.8 kV was applied to the platinum wire inserted in solution in the tip. The inlet capillary of the MS was heated to 200 ^o^C, S-lens RF level was set at 100, automatic gain control target set at 5 × 10^6^ with a maximum injection time of 200 ms.

### Digestion and MS/MS analysis

WbbB_GT99_-E158Q was reacted with the CMP-Kdo reaction mix, or WbbB-WT was reacted with the CMP-Kdo reaction mix followed by NaBH_4_, as described above. A fresh solution of pepsin from porcine stomach (V195A, Promega, Madison, WI, USA) was then added to the WbbB sample dissolved in 200 mM aqueous ammonium acetate (pH 3.5) at a pepsin:WbbB ratio of 1:20, and the solution was incubated for 1 hr at 37 ^o^C. The pepsin digest solution (3-5 μL) was placed into nanoESI glass tip and ESI mass spectra of the digest were obtained using a Q Exactive Orbitrap (Orbitrap) mass spectrometer equipped with nanoESI source. The nanoESI spray voltage of ~0.8 kV was applied to the platinum wire inserted in solution in the tip. The inlet capillary of the MS was heated to 200 ^o^C, S-lens RF level was set at 50, automatic gain control target set at 5 × 10^6^ with a maximum injection time of 200 ms. The MS/MS analysis of the WbbB_GT99_-E158Q sample was performed by isolating the precursor ions at *m/z* 1139.46 (isolation width of 0.4 Th) and fragmentation by higher-energy collisional dissociation (HCD) using nitrogen gas and collision energies (CE) ranging from 30 V to 50 V. The MS/MS analysis of the NaBH_4_ treated WbbB_GT99_-WT sample was performed by isolating the precursor ions at *m/z* 905.42 or at *m/z* 1125.48 (isolation width of 0.4 Th) and fragmentation by HCD using nitrogen gas at CE ranging from 30 V to 50 V. All MS data were acquired and processed using Thermo Xcalibur 4.1 software. MS/MS fragments were annotated using the MS-Product tool in UCSF ProteinProspector v 6.4.0 software.

### Crystallization, substrate soaks, and structure determination

Protein was concentrated to 5–15 mg/mL (in a buffer containing 20 mM Tris, 150 mM NaCl and 5 mM 2-mercaptoethanol) using 10 kDa molecular weight cut-off Amicon Ultra Centrifugal Filters (Merck) and supplemented with 5 mM CMP (Bio Basic). Crystallization experiments were conducted in a sitting-drop configuration at room temperature, with protein mixed (in 2:1 or 1:1 ratio with well solution) and equilibrated against ~80 μL of well solution. Prismatic crystals generally grew within 7 days, except the acceptor **2** complexes, which grew within 5 weeks. After necessary manipulations, all crystals were cryoprotected with paratone-N oil before freezing in liquid nitrogen for data collection at 100 K.

For the WbbB_GT99_-D232N + CMP-β-Kdo complex structure, crystals were grown with a well solution containing 100 mM NaCl, 100 mM HEPES pH 7.5, 1.6 M ammonium sulfate. A CMP-β-Kdo generating reaction mixture was prepared containing 100 mM NaCl, 100 mM HEPES pH 7.5, 1.6 M ammonium sulfate, 10 mM MgCl_2_, 4 mM Kdo, 5 mM CTP, and 2.5 µg KdsB. Crystals were transferred from wells into a droplet of reaction mixture and soaked for 30 min prior to freezing.

For the WbbB_GT99_-D232N-Kdo adduct complex structure, crystals were grown using a well condition containing 1.1 M sodium malonate, 100 mM HEPES, and 0.5% Jeffamine ED-2001 pH 7.0. A CMP-β-Kdo-generating reaction solution was prepared in a similar buffer (43 mM HEPES pH 7.0, 1.4 M sodium malonate pH 7.0, 8.6 mM MgCl_2_, 8.6 mM CTP, 8.6 mM Kdo and 2 μg of KdsB) and incubated for 10 min at 37 °C. After removal of most of the original mother liquor, the crystal was washed 3 times with the reaction mix, then incubated for 5 min at room temperature prior to harvesting.

The WbbB_GT99_-D232C-Kdo-adduct crystals were grown from a well solution containing 200 mM ammonium sulfate, 100 mM Bis-Tris pH 6.5, 25% (w/v) PEG 3350. A reaction mix was prepared containing 200 mM ammonium sulphate, 100 mM Bis-Tris pH 6.5, 25% PEG 3350, 10 mM MgCl_2_, 2 mM Kdo, 5 mM CTP and 5 μg of KdsB. Crystals were transferred into this solution for approximately 5 min, and then frozen in paratone oil.

The WbbB_GT99_ acceptor **2** complex crystals were grown from protein supplemented with 5 mM acceptor **2** (from a 26 mM stock dissolved in DMSO) and crystallized using 1.1 M sodium malonate, 100 mM HEPES, and 0.5% Jeffamine ED-2001, pH 7.0. This structure also has three additional weak acceptor binding sites (partial occupancy and elevated ADPs) which are removed from the active site and generally at crystal packing interfaces (Supplementary Fig. 10a, b); occupancy of these sites are likely artifacts of the high substrate concentration used in crystallization.

For the WbbB_GT99_-D232C ternary complex, protein expressed in *E. coli* BL21(DE3) was supplemented with 5 mM acceptor **2** (from a 54 mM stock in DMSO) and crystallized using a well solution containing 1.1 M sodium malonate, 100 mM HEPES, and 0.5% Jeffamine ED-2001, pH 7.0.

Data was collected at the Canadian Light Source Beam Line ID1 (D232N-CMP-Kdo and D232C-Kdo) or BM1 (D232N-Kdo, wt_acceptor, and D232C-ternary). Data was processed and scaled using the XDS package^44^. All structures were determined using molecular replacement in Phaser in Phenix^45^, utilizing chain A of 5FA1 as a search model. Rebuilding was performed in Coot^46^, with refinement in Phenix.refine^47^. Data collection and structure refinement statistics are shown in Table S2. All structure figures were prepared using Pymol v2.0 (Schrödinger LLC).

## Supplementary information

Supplementary information

Description of Additional Supplementary files

Supplementary movie 1

## Data Availability

Source data for Supplemental Figs. 1c, 3a, b (as reflected in Table 1) are provided with this paper. The structures of WbbB_GT99_ have been deposited at the protein databank as 8CSB (D232N CMP-β-Kdo complex), 8CSC (D232N-Kdo adduct), 8CSD (D232C-Kdo adduct), 8CSE (wild type acceptor complex) and 8CSF (D232N-Kdo ternary complex). Other data that support this study are available from the corresponding author upon reasonable request. Source data are provided with this paper.

## Source data

Source Data

## References

[1] Lairson LL, Henrissat B, Davies GJ, Withers SG (2008). Glycosyltransferases: structures, functions, and mechanisms. Annu. Rev. Biochem.

[2] Lombard V, Golaconda Ramulu H, Drula E, Coutinho PM, Henrissat B (2014). The carbohydrate-active enzymes database (CAZy) in 2013. Nucleic Acids Res..

[3] Koshland DE (1953). Sterochemistry and the mechanism of enzymatic reactions. Biol. Rev..

[4] Yip VLY, Withers SG (2004). Nature’s many mechanisms for the degradation of oligosaccharides. Org. Biomol. Chem..

[5] Vocadlo DJ, Davies GJ, Laine R, Withers SG (2001). Catalysis by hen egg-white lysozyme proceeds via a covalent intermediate. Nature.

[6] Lairson LL (2004). Intermediate trapping on a mutant retaining alpha-galactosyltransferase identifies an unexpected aspartate residue. J. Biol. Chem..

[7] Soya N, Fang Y, Palcic MM, Klassen JS (2011). Trapping and characterization of covalent intermediates of mutant retaining glycosyltransferases. Glycobiology.

[8] Rojas-Cervellera V, Ardèvol A, Boero M, Planas A, Rovira C (2013). Formation of a covalent glycosyl-enzyme species in a retaining glycosyltransferase. Chemistry.

[9] Blackler RJ (2017). Glycosyltransfer in mutants of putative catalytic residue Glu303 of the human ABO(H) A and B blood group glycosyltransferases GTA and GTB proceeds through a labile active site. Glycobiology.

[10] Ly HD, Lougheed B, Wakarchuk WW, Withers SG (2002). Mechanistic studies of a retaining α-Galactosyltransferase from *Neisseria meningitidis*. Biochemistry.

[11] Ardèvol A, Iglesias-Fernández J, Rojas-Cervellera V, Rovira C (2016). The reaction mechanism of retaining glycosyltransferases. Biochem. Soc. Trans..

[12] Yu H (2015). Notch-modifying xylosyltransferase structures support an SNi-like retaining mechanism. Nat. Chem. Biol..

[13] Gómez H, Polyak I, Thiel W, Lluch JM, Masgrau L (2012). Retaining glycosyltransferase mechanism studied by QM/MM methods: lipopolysaccharyl-α−1,4-galactosyltransferase C transfers α-galactose via an oxocarbenium ion-like transition state. J. Am. Chem. Soc..

[14] Bobovská A, Tvaroška I, Kóňa J (2014). A theoretical study on the catalytic mechanism of the retaining α−1,2-mannosyltransferase Kre2p/Mnt1p: the impact of different metal ions on catalysis. Org. Biomol. Chem..

[15] Ardèvol A, Rovira C (2011). The molecular mechanism of enzymatic glycosyl transfer with retention of configuration: evidence for a short-lived oxocarbenium-like species. Angew. Chem. Int Ed. Engl..

[16] Trnka T, Kozmon S, Tvaroška I, Koča J (2015). Stepwise catalytic mechanism via short-lived intermediate inferred from combined QM/MM MERP and PES calculations on retaining glycosyltransferase ppGalNAcT2. PLoS Comp. Biol..

[17] Paparella, A. S., Cahill, S. M., Aboulache, B. L. & Schramm, V. L. Clostridioides difficile TcdB Toxin Glucosylates Rho GTPase by an SNi Mechanism and Ion Pair Transition State. *ACS Chem. Biol*. 10.1021/acschembio.2c00408 (2022). 10.1021/acschembio.2c00408 PMC9486934 36038138

[18] Ferreira, P., Fernandes, P. A. & Ramos, M. J. The catalytic mechanism of the retaining glycosyltransferase mannosylglycerate synthase. **27**, 13998–14006 (2021). 10.1002/chem.202101724 34355437

[19] Gómez H, Mendoza F, Lluch JM, Masgrau L (2015). QM/MM studies reveal how substrate-substrate and enzyme-substrate interactions modulate retaining glycosyltransferases catalysis and mechanism. Adv. Protein Chem. Struct. Biol..

[20] Lee SS (2011). Mechanistic evidence for a front-side, SNi-type reaction in a retaining glycosyltransferase. Nat. Chem. Biol..

[21] Ovchinnikova, O. G. et al. Bacterial β-Kdo glycosyltransferases represent a new glycosyltransferase family (GT99). **113**, E3120–E3129 (2016). 10.1073/pnas.1603146113 PMC4896679 27199480

[22] Williams DM (2017). Single polysaccharide assembly protein that integrates polymerization, termination, and chain-length quality control. Proc. Natl Acad. Sci. USA.

[23] Mann E, Mallette E, Clarke BR, Kimber MS, Whitfield C (2016). The Klebsiella pneumoniae O12 ATP-binding Cassette (ABC) Transporter Recognizes the Terminal Residue of Its O-antigen polysaccharide substrate. J. Biol. Chem..

[24] Ovchinnikova OG (2016). Biochemical characterization of bifunctional 3-Deoxy-β-d-manno-oct-2-ulosonic Acid (β-Kdo) Transferase KpsC from Escherichia coli involved in capsule biosynthesis. J. Biol. Chem..

[25] Doyle L (2019). Biosynthesis of a conserved glycolipid anchor for Gram-negative bacterial capsules. Nat. Chem. Biol..

[26] Ni L (2007). Crystal structures of *Pasteurella multocida* sialyltransferase complexes with acceptor and donor analogues reveal substrate binding sites and catalytic mechanism. Biochemistry.

[27] Lin LYC (2011). Structure and mechanism of the lipooligosaccharide sialyltransferase from Neisseria meningitidis. J. Biol. Chem..

[28] Lin CH, Murray BW, Ollmann IR, Wong CH (1997). Why is CMP-ketodeoxyoctonate highly unstable?. Biochemistry.

[29] Kohlbrenner WE, Fesik SW (1985). Determination of the anomeric specificity of the Escherichia coli CTP:CMP-3-deoxy-D-manno-octulosonate cytidylyltransferase by 13C NMR spectroscopy. J. Biol. Chem..

[30] Mamat U (2015). Detoxifying Escherichia coli for endotoxin-free production of recombinant proteins. Microb. Cell Factories.

[31] Eshdat Y, Dunn A, Sharon N (1974). Chemical conversion of aspartic acid 52, a catalytic residue in hen egg-white lysozyme, to homoserine. Proc. Natl Acad. Sci. USA.

[32] McGregor NGS (2021). Cysteine nucleophiles in glycosidase catalysis: application of a covalent β-l-Arabinofuranosidase inhibitor. Angew. Chem. Int Ed. Engl..

[33] Wilson JC, Kiefel MJ, Angus DI, von Itzstein M (1999). Investigation of the stability of thiosialosides toward hydrolysis by sialidases using NMR spectroscopy. Org. Lett..

[34] Agirre J (2017). Strategies for carbohydrate model building, refinement and validation. Acta Crystallogr. Sect. D. Struct. Biol..

[35] Davies GJ (1998). Snapshots along an enzymatic reaction coordinate: Analysis of a retaining beta-glycoside hydrolase. Biochemistry.

[36] Wild R (2018). Structure of the yeast oligosaccharyltransferase complex gives insight into eukaryotic N-glycosylation. Science.

[37] Mark BL, James M (2002). Anchimeric assistance in hexosaminidases. Can. J. Chem.-Rev. Canadienne De. Chim..

[38] Ottiger P (2009). Strong N-H…pi hydrogen bonding in amide-benzene interactions. J. Phys. Chem. B.

[39] Horenstein BA, Bruner M (1998). 1998. The N-Acetyl neuraminyl oxecarbenium ion is an intermediate in the presence of anionic nucleophiles. J. Am. Chem. Soc..

[40] Chiu CPC (2004). Structural analysis of the sialyltransferase CstII from *Campylobacter jejuni* in complex with a substrate analog. Nat. Struct. Mol. Biol..

[41] Moremen KW, Haltiwanger RS (2019). Emerging structural insights into glycosyltransferase-mediated synthesis of glycans. Nat. Chem. Biol..

[42] Bryant FR (1988). Construction of a recombinase-deficient mutant recA protein that retains single-stranded DNA-dependent ATPase activity. J. Biol. Chem..

[43] Studier FW, Moffatt BA (1986). Use of bacteriophage T7 RNA polymerase to direct selective high-level expression of cloned genes. J. Mol. Biol..

[44] Kabsch W (2010). XDS. Acta. Crystallogr. D. Biol. Crystallogr.

[45] McCoy AJ (2007). Phaser crystallographic software. J. Appl Crystallogr.

[46] Emsley P, Cowtan K (2004). Coot: model-building tools for molecular graphics. Acta. Crystallogr. D. Biol. Crystallogr..

[47] Adams PD (2002). PHENIX: building new software for automated crystallographic structure determination. Acta. Crystallogr D. Biol. Crystallogr.

[48] Mirdita M (2022). ColabFold: making protein folding accessible to all. Nat. Methods.

