# Author Correction: The retaining β-Kdo glycosyltransferase WbbB uses a double-displacement mechanism with an intermediate adduct rearrangement step

**DOI:** 10.1038/s41467-022-35491-z

**Published:** 2022-12-12

**Authors:** Taylor J. B. Forrester, Olga G. Ovchinnikova, Zhixiong Li, Elena N. Kitova, Jeremy T. Nothof, Akihiko Koizumi, John S. Klassen, Todd L. Lowary, Chris Whitfield, Matthew S. Kimber

**Affiliations:** 1 Department of Molecular and Cellular Biology, University of Guelph, 50 Stone Road E., Guelph, ON N1G 2W1 Canada; 2 Department of Chemistry, University of Alberta, 11227 Saskatchewan Drive, Edmonton, AB T6G 2G2 Canada; 3 Institute of Biological Chemistry, Academia Sinica, Academia Road, Section 2, #128, Nangang, Taipei, 11529 Taiwan; 4 Institute of Biochemical Sciences, National Taiwan University, Section 4, #1, Roosevelt Road, Taipei, 10617 Taiwan

**Keywords:** Glycobiology, X-ray crystallography, Enzyme mechanisms

Correction to: *Nature Communications* 10.1038/s41467-022-33988-1, published online 21 October 2022

The original version of this Article contained an error in the introduction, which incorrectly stated ‘Quantum mechanics/molecular mechanics (QM/MM) simulations of this enzyme do indicate participation of Glu303 in the mechanism, but the covalent-like interaction is highly transitory, lasting only for only a few picoseconds^8^ and mutation of this residue to cysteine or aspartate only slightly slows the reaction^9^.’ The correct version states ‘Quantum mechanics/molecular mechanics (QM/MM) metadynamics analysis of a bovine GT-6’s mechanism predicts that, while the substrates are organized very similarly to other retaining GTs, the donor saccharide forms an intermediate glutamate-galactose covalent adduct prior to transfer to the acceptor^8^; however, in later experiments on human GT-6, mutation of Glu303 to cysteine or aspartate was found to only slightly slow the reaction^9^.’

This has been corrected in both the PDF and HTML versions of the Article.

