# Supplementary material for: The retaining β-Kdo glycosyltransferase WbbB uses a double-displacement mechanism with an intermediate adduct rearrangement step
Source: Nat Commun. 2022 Oct 21;13:6277. doi: 10.1038/s41467-022-33988-1 (PMC9587256; doi:10.1038/s41467-022-33988-1)
Supplement: Supplementary file 1 — Supplementary information [file 41467_2022_33988_MOESM1_ESM.pdf]

Supplementary Information

Supplementary Figures and Tables

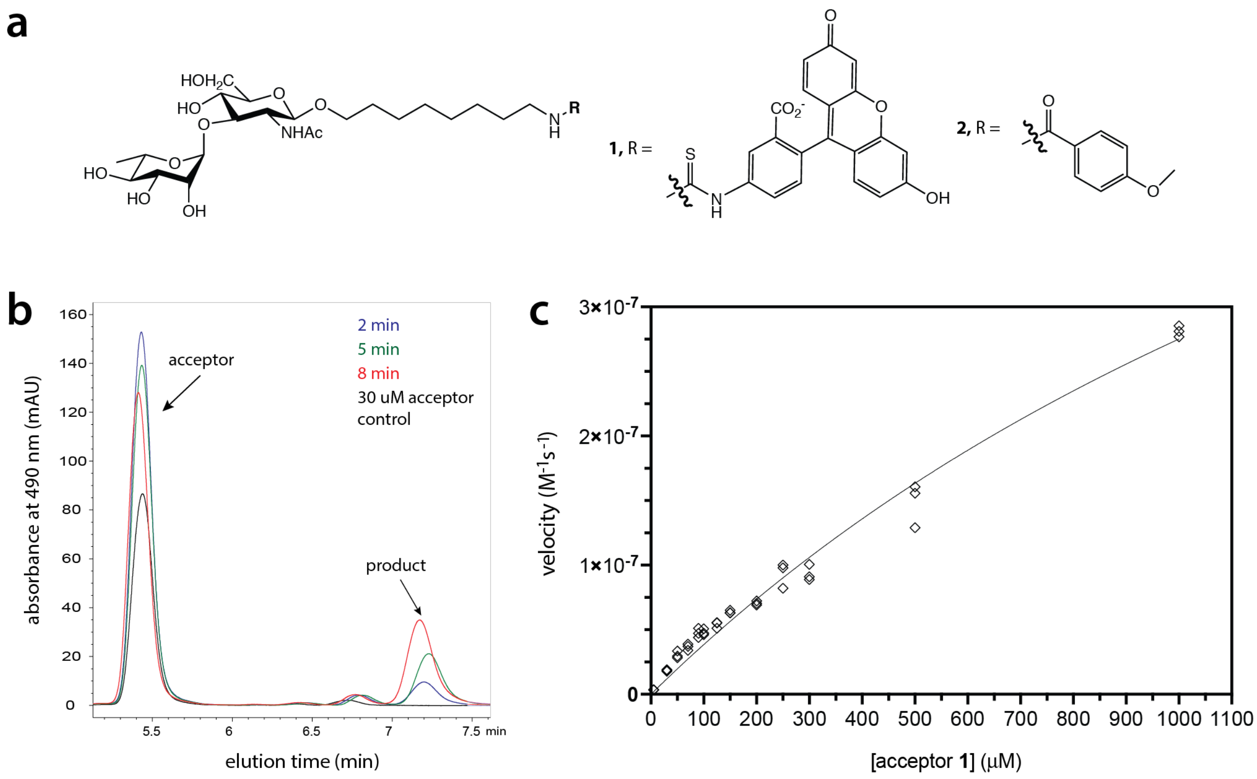

Supplementary Figure 1.
Synthetic acceptors and kinetics assays. a) Chemical structures of acceptors used in this study. b) HPLC traces for a single assay replicate at 100 μM acceptor **1**. The relative quantities of acceptor and product were measured at each time point by integrating under the respective curves. A trace of unreacted acceptor (at 30 μM) is shown as a control. c) Initial velocity vs substrate concentration plot for acceptor **1**. The line traces best fit. Acceptor **1** does not show substrate saturation at 1 mM. Three technical replicates are shown for each concentration of acceptor **1** tested. Source data are provided as a Source Data file.

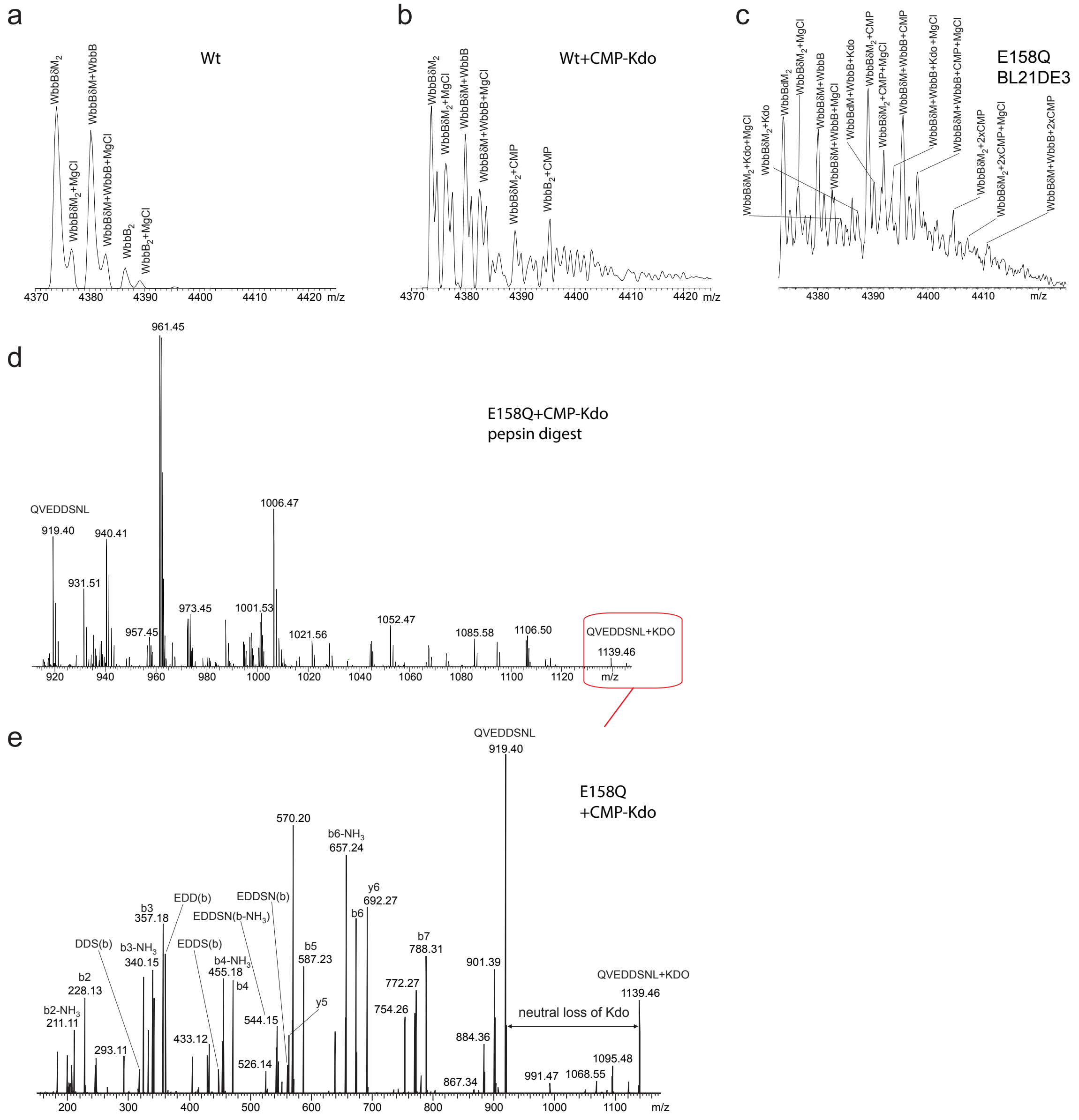

Supplementary Figure 2.

Supplementary MS data. ESI mass spectra were acquired with a UHMR Orbitrap mass spectrometer (a-c) from aqueous ammonium acetate solutions (200 mM, pH 7, and 25 °C) and represent the +21 charge state of the dimer of $WbbB_{GT99}$ variants.

a) Unreacted $WbbB_{GT99}$-wildtype (5 µM) purified from BL21(DE3). b) Mass spectrum of $WbbB_{GT99}$-wildtype after reaction with CMP-Kdo in presence of $MgCl_2$. c) ESI mass spectrum for $WbbB_{GT99}$-E158Q (5 µM) expressed in BL21(DE3). d) ESI mass spectrum acquired with a Q Exactive Orbitrap mass spectrometer of $WbbB_{GT99}$-E158Q after reaction with CMP-Kdo and subsequent pepsin digest. e) $WbbB_{GT99}$-E158Q was reacted with CMP-Kdo, digested with pepsin; a MS/MS spectrum of the singly charged precursor ion (*m/z* 1139.46) of the ($Q_{228}VEDDSNL_{235}$+Kdo) peptide was then acquired with a Q Exactive Orbitrap mass spectrometer at collision energy (CE) of 50 V. Source data are provided as a Source Data file.

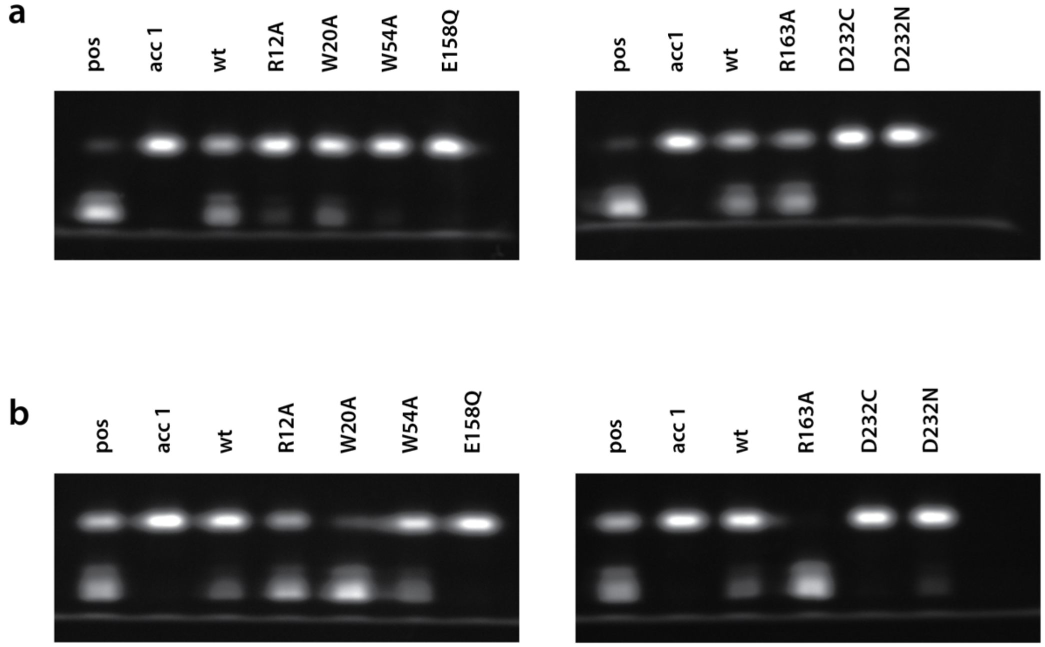

Supplementary Figure 3.
SDS PAGE analysis of $WbbB_{GT99}$ variant reactions. a) Reactions where the protein variants were added at 5 µg $WbbB_{GT99}$ variants. b) Reactions where protein variants were incubated with 50 µg $WbbB_{GT99}$ variants. For both sets, acceptor **1** was incubated with a CMP-β-Kdo generating reaction mix and the identified variants for 8 minutes, after which the reaction was stopped, the reaction mix was run on an SDS PAGE gel and the resulting fluorescence analyzed in ImageJ. Under these conditions 0.5 µg of wild-type $WbbB_{GT99}$ does not go to completion. Note that the addition of a second negative charge to the acceptor makes it migrate faster under these conditions. pos – positive control where wild-type enzyme was added and the reaction allowed to run to near completion. acc **1** – acceptor **1**; wt – wild type $WbbB_{GT99}$, added at 0.5 µg/ml. Other enzyme variants are as labelled. Source data are provided as a Source Data file. For a) variants were evaluated from four replicates while for b) variants were evaluated from five replicates.

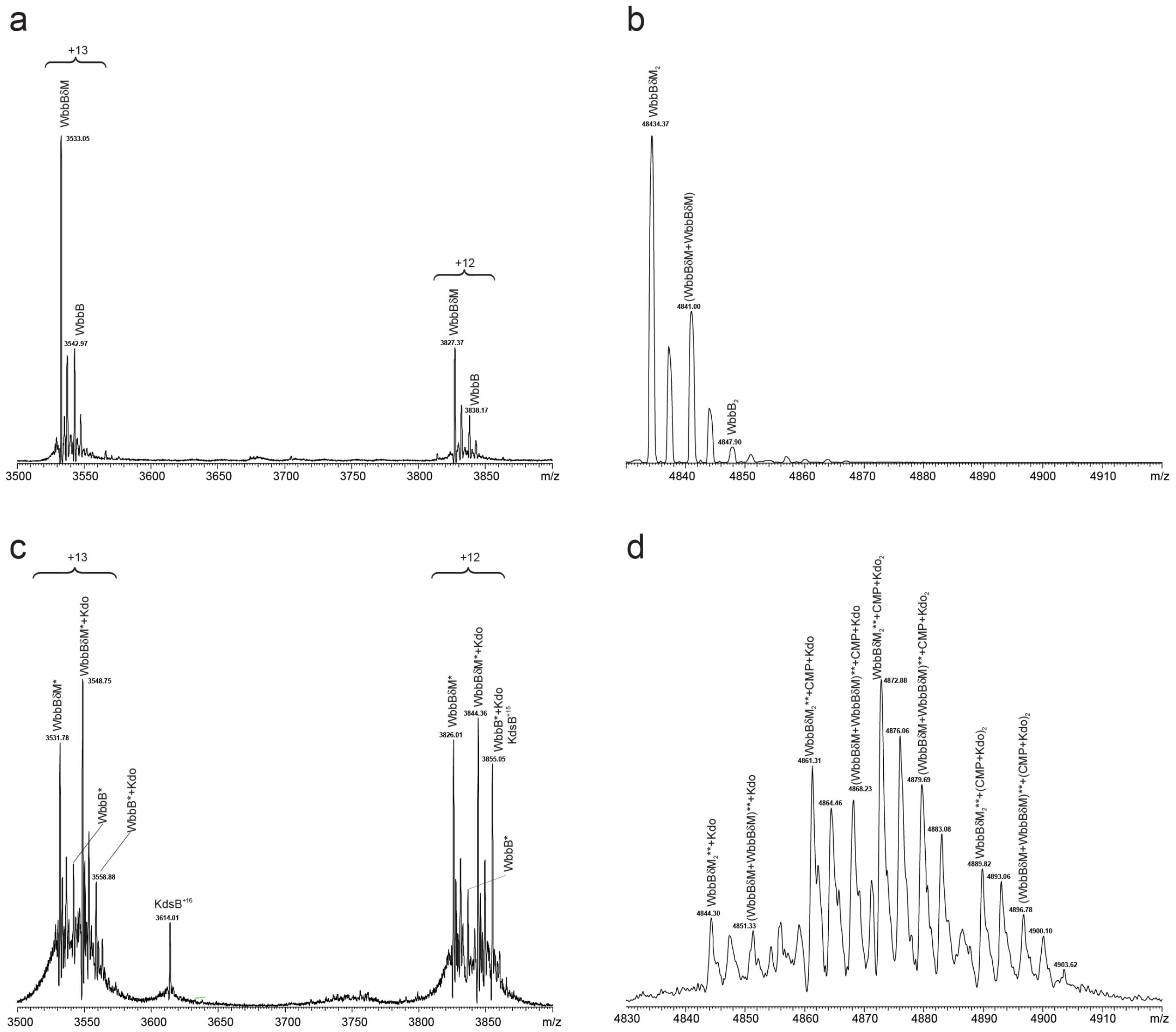

Supplementary Figure 4.

Sodium borohydride reduction of WbbB$_{GT99}$-wildtype Kdo adducts**.** Native ESI mass spectra of the a) monomer and b) dimer of WbbB$_{GT99}$-wildtype in the absence of the CMP-Kdo generating mix. c) Native MS evidence for the formation of homoserine (HSE) derivative of a WbbB$_{GT99}$-wildtype monomer after reduction of the covalent Kdo intermediate with sodium borohydride. Ion peaks corresponding to +12 and +13 charge states of the WbbB$_{GT99}$-wildtype monomer species are shown. * indicates that molecular weight of is shifted by 14 Da, corresponding to a single Asp to Hse (homoserine) conversion. d) Ion peaks corresponding to +19 charge state of the WbbB$_{GT99}$-wildtype dimer species after reduction of the covalent Kdo intermediate with sodium borohydride. ** indicates that molecular weight of WT dimer species is shifted by 28 Da, corresponding to two Asp to Hse (homoserine) conversions (seen only in the WbbB$_{GT99}$ dimer).

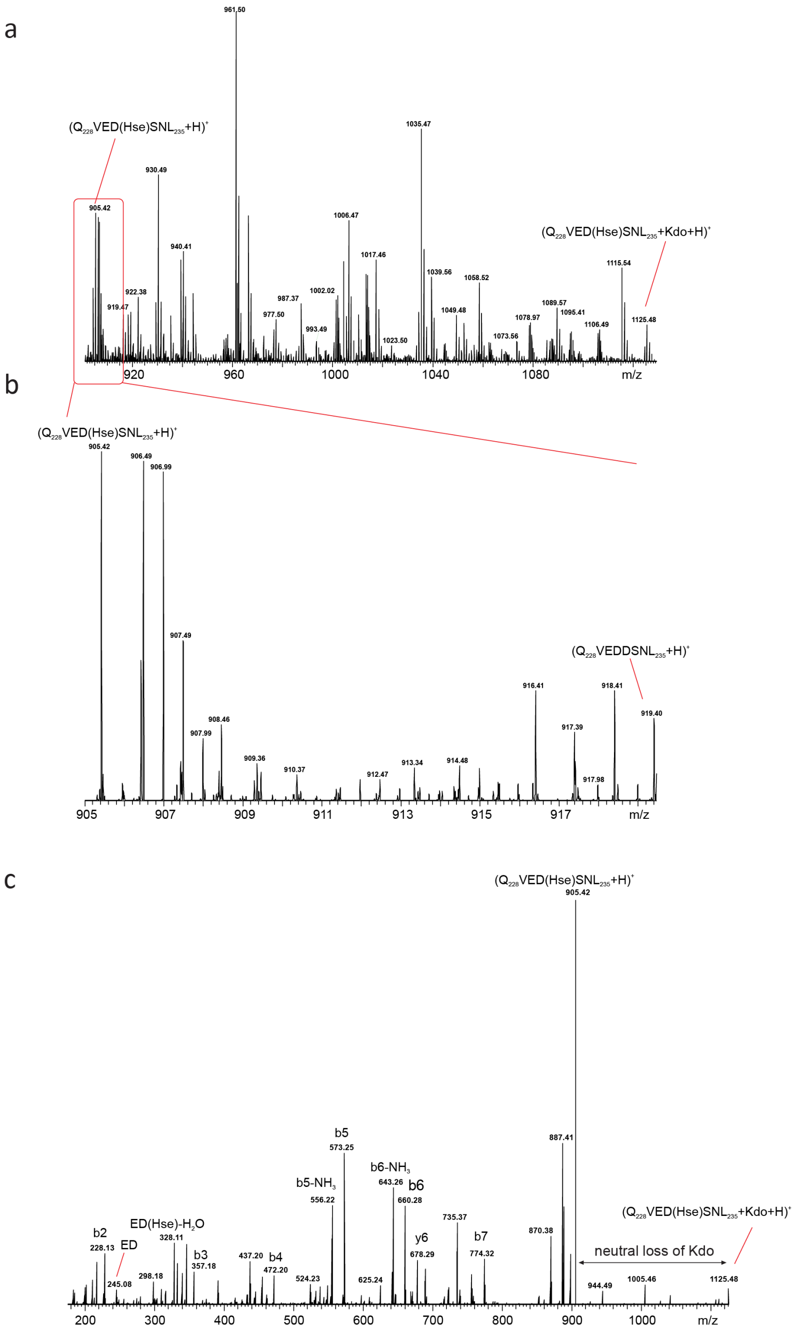

Supplementary Figure 5.

Representative mass spectra of WbbB-wildtype pepsin digest after reaction with CMP-Kdo and reduction using sodium borohydride. a) Digestion with pepsin results in peptides showing Asp to homoserine conversion as both free peptides and as Kdo adducts (the (QVED(Hse)SNL+Kdo+H)$^+$ ion signal, where Hse = homoserine). b) Details of the region from 905 – 920 m/z. In addition to the homoserine containing peptide, a minor unmodified species is also detected as (QVEDDSNL+H)$^+$ ions. c) MS/MS spectrum of the singly charged peptide (Q$_{228}$VED(Hse)SNL$_{235}$)+Kdo) precursor ion (m/z 1125.48) acquired with a Q Exactive Orbitrap mass spectrometer at collision energy (CE) of 40 V. Analysis of the % conversion to Hse are provided as a Source Data file.

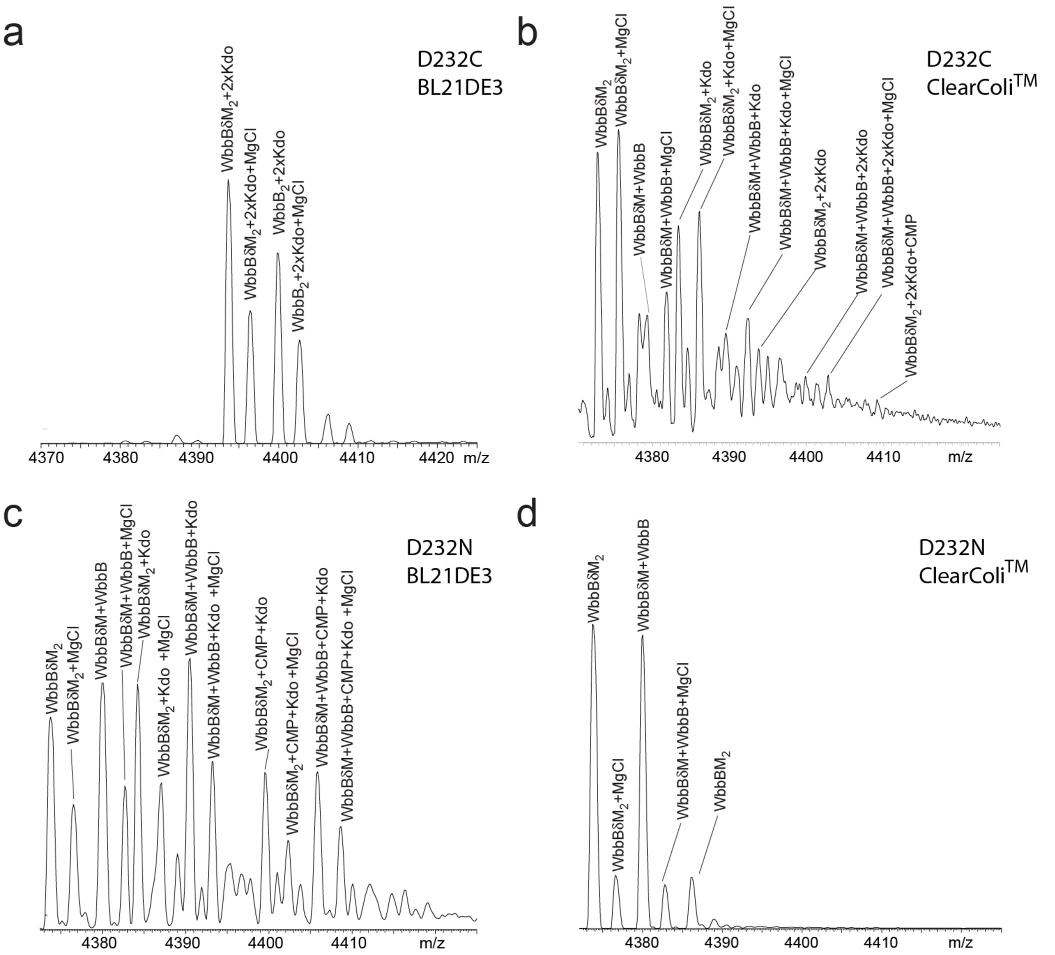

Supplementary Figure 6.
Supplementary MS data showing Kdo adduct formation of WbbB$_{GT99}$ variants during expression in different *E. coli* strains. All ESI mass spectra were acquired with a UHMR Orbitrap mass spectrometer, and were collected for aqueous ammonium acetate solutions (200 mM, pH 7, and 25 °C) and represent the +21 charge state of the dimer of WbbB$_{GT99}$ variants.
a) WbbB$_{GT99}$-D232C expressed in BL21(DE3). b) WbbB$_{GT99}$-D232C expressed in ClearColi™.
c) WbbB$_{GT99}$-D232N expressed in BL21(DE3). d) WbbB$_{GT99}$-D232N expressed in ClearColi™.

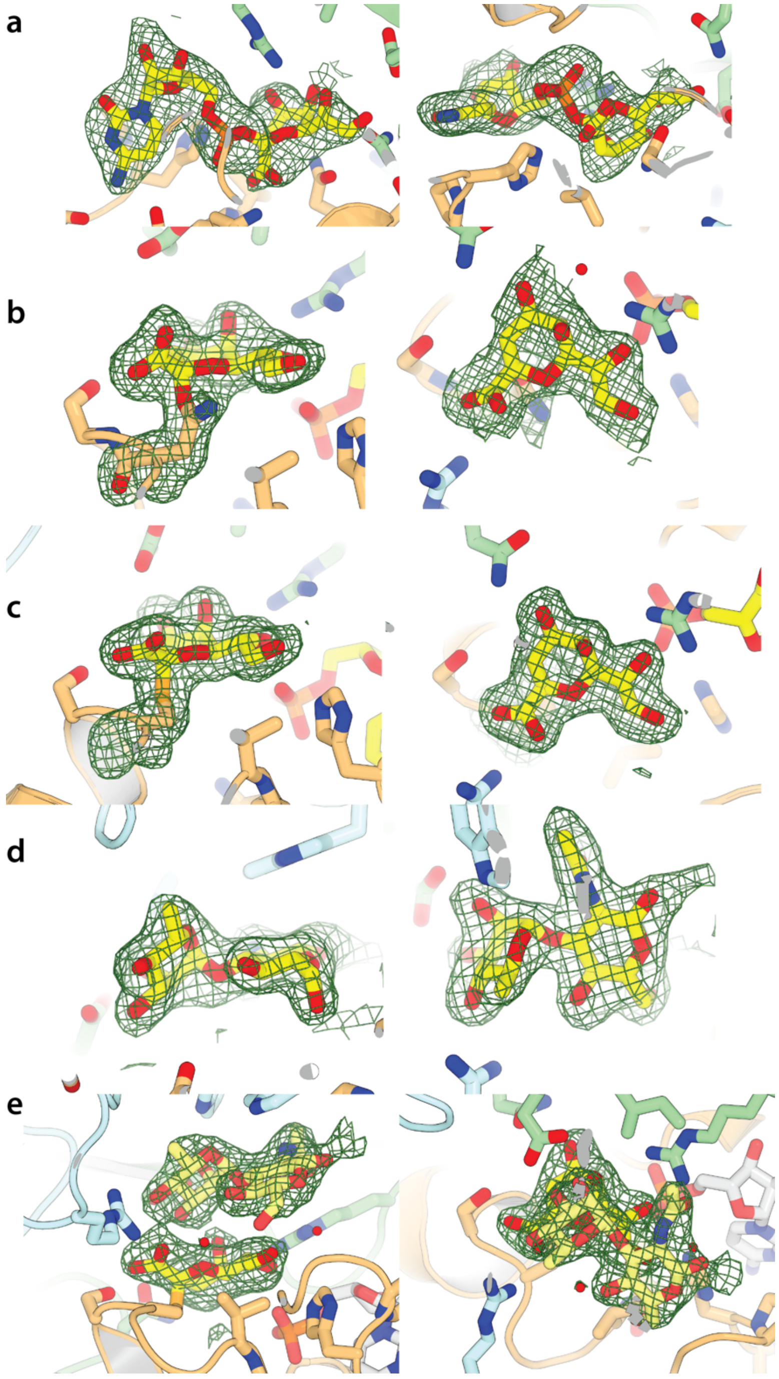

Supplementary Figure 7.
Omit maps for ligands in the ligand co-structures. Polder omit maps were calculated for all ligands. Two orthogonal views are shown for all structures. a) $WbbB_{GT99}$ D232N CMP-Kdo complex with CMP-Kdo polder map shown at 3.0 σ in green. b) $WbbB_{GT99}$ D232N-Kdo adduct. Polder map for Asn232-Kdo shown at 4.0 σ in green. c) $WbbB_{GT99}$ D232C-Kdo adduct. Polder map for Cys232-Kdo shown at 4.0 σ in green. d) $WbbB_{GT99}$ acceptor complex. Polder map for acceptor **2** disaccharide is shown at 3.0 σ in green. e) $WbbB_{GT99}$ D232C-Kdo adduct plus acceptor complex. Polder map for D232C-Kdo adduct and acceptor **2** shown at 4.0 σ in green.

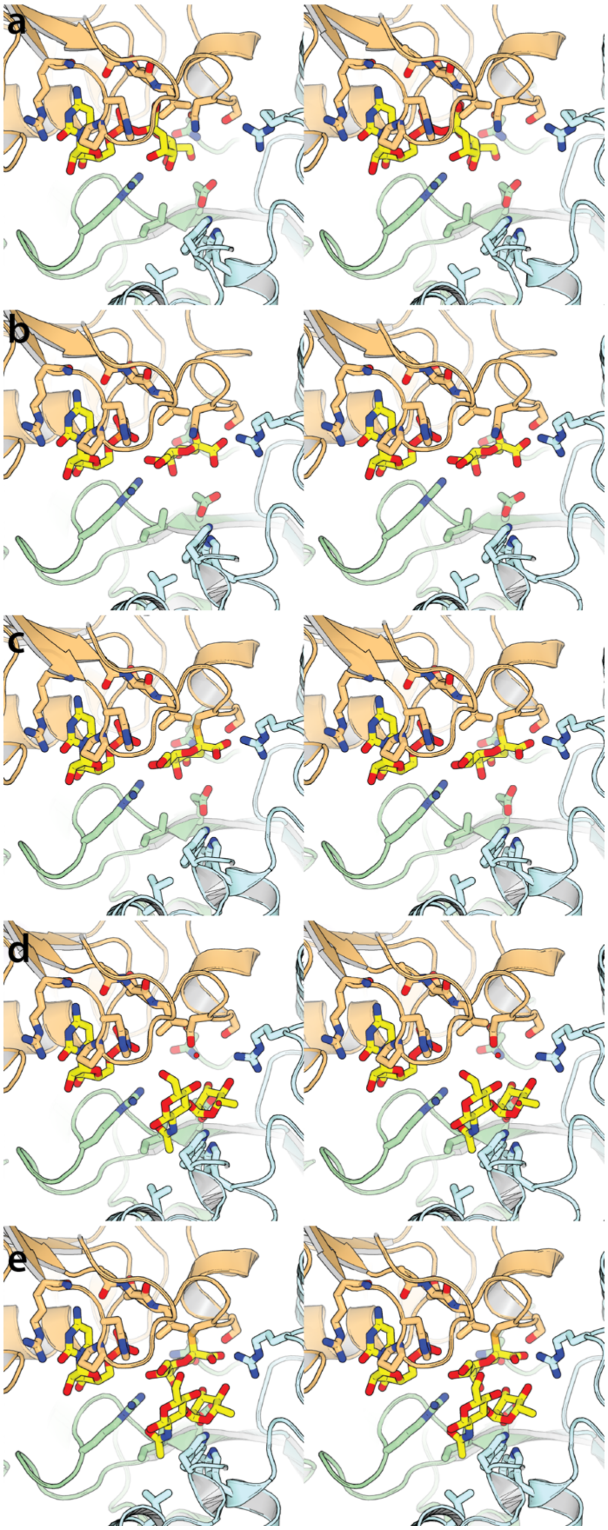

Supplementary Figure 8.
Wall-eyed stereo figures showing all catalytic complexes shown from a consistent viewpoint. Colouring and other conventions are as in the main text figures. a) $\mathrm{WbbB_{GT99}}$ D232N CMP-Kdo complex. b) $\mathrm{WbbB_{GT99}}$ D232N-Kdo adduct. c) $\mathrm{WbbB_{GT99}}$ D232C-Kdo adduct. d) $\mathrm{WbbB_{GT99}}$ acceptor **2** complex. e) $\mathrm{WbbB_{GT99}}$ D232C-Kdo adduct plus acceptor **2** complex.

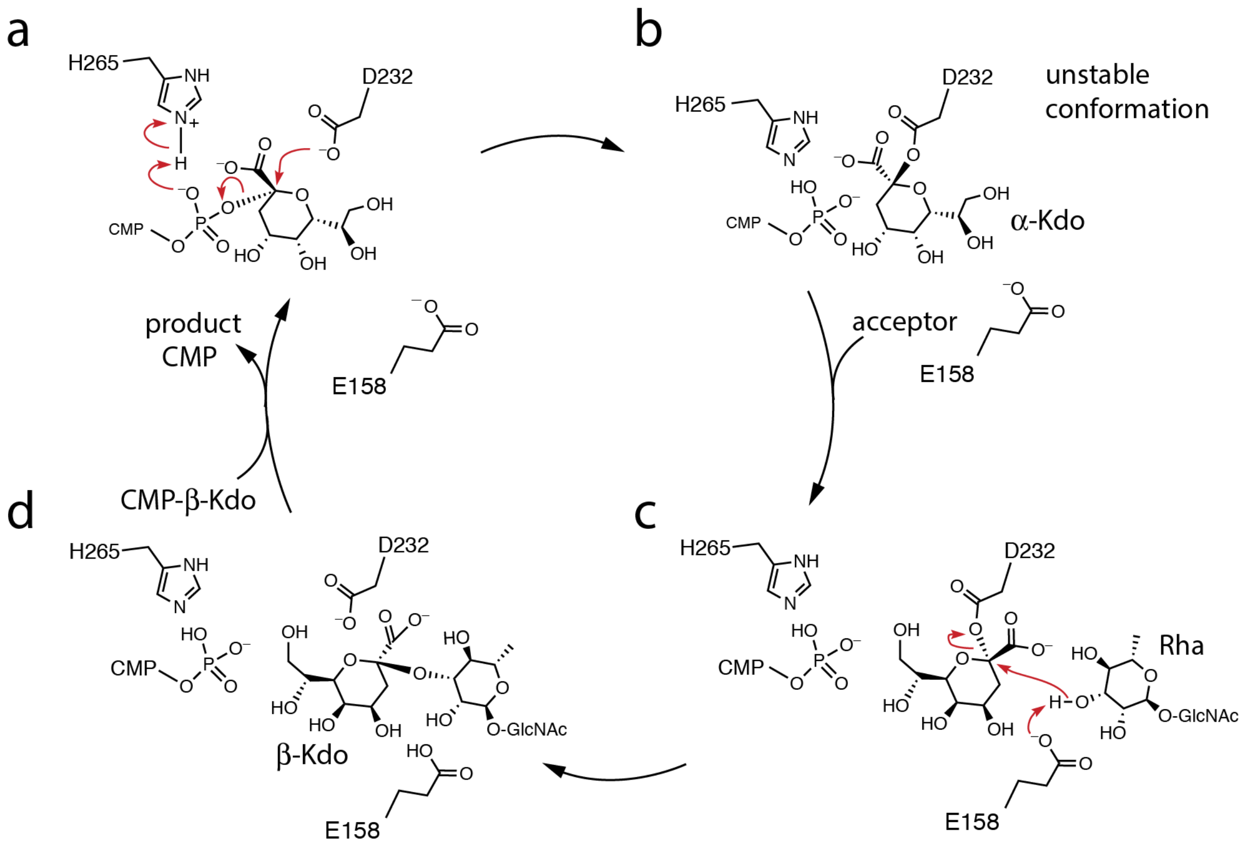

Supplementary Figure 9.

Proposed mechanism for $WbbB_{GT99}$ retaining β-Kdo transferase. a) CMP-β-Kdo binds in the active site. Asp232 carboxylate performs a nucleophilic attack on the anomeric carbon, while a proton is transferred from His265 to the leaving phosphate group. b) Immediate product of the first half reaction. This conformation has not been directly observed and is assumed to be unstable and short-lived. c) Reorganized Asp232–α-Kdo intermediate complex. While the acceptor can bind to the empty active site, the additional interactions mediated by Asp232–α-Kdo imply that this state has significantly higher affinity. The O3 hydroxyl group, activated by the general base Glu158, attacks the anomeric carbon of Kdo in a second inverting reaction, with Asp232 as the leaving group. d) Product of the second reaction; this complex also appears to be unstable, most likely due to the inversion of the anomeric configuration of Kdo, as well as possible close contacts with Asp232, and has not been directly observed.

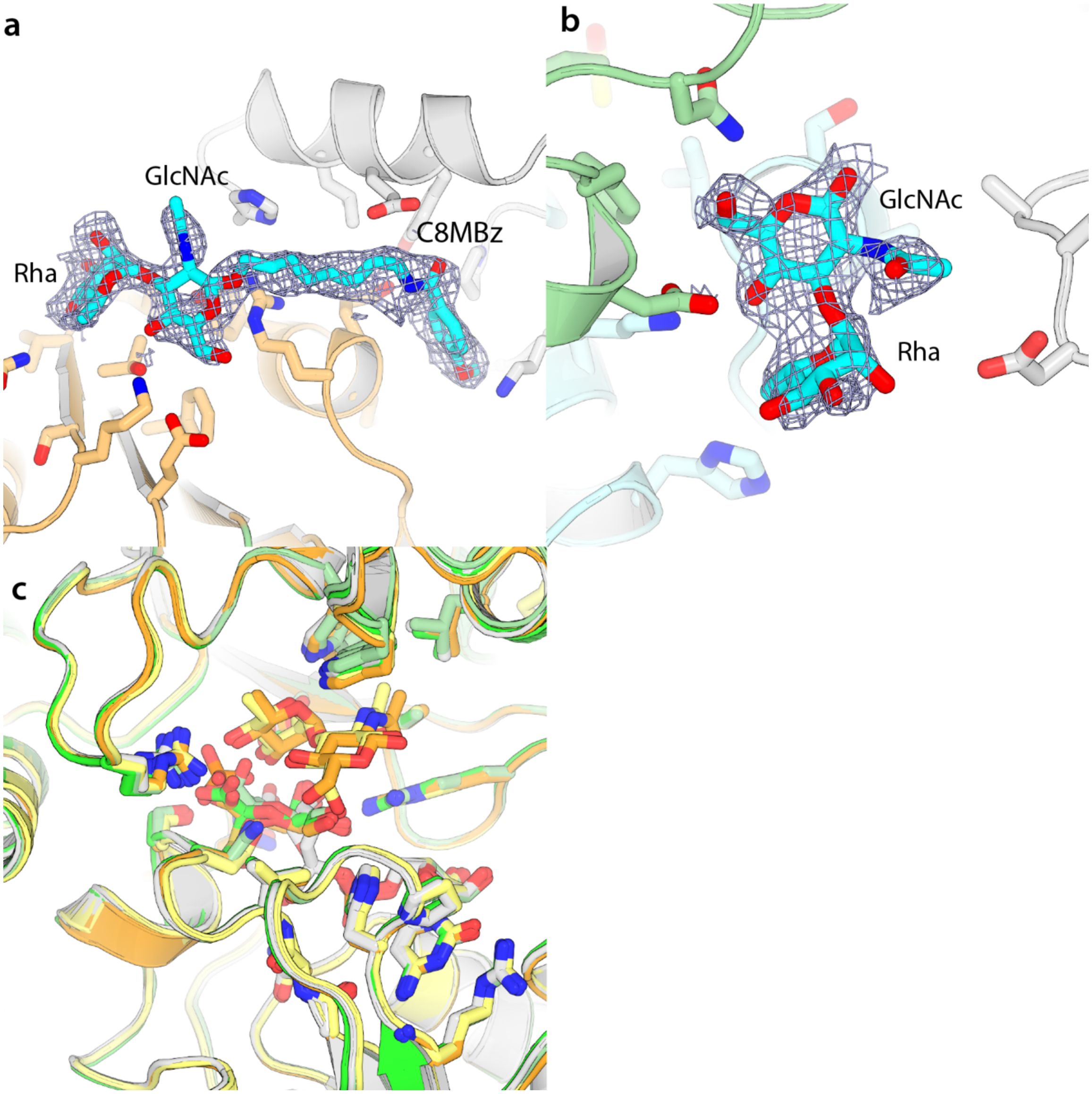

Supplementary Figure 10.
Additional structure figures. a) Electron density showing weak acceptor binding in a non-biological site. Electron density mesh represents the 2mFo-DFc map contoured at 0.5 σ. Residues in white are contributed by a crystal symmetry mate. C8MBz is the C8-methoxybenzamidine aglycone. b) The second low occupancy binding site. Electron density is contoured at 0.8 σ. This site is also similarly occupied in the second chain in the asymmetric unit. c) Superpositions of the various $WbbB_{GT99}$ complexes. The D232N CMP−β-Kdo complex is shown in white; the D232N-Kdo adduct structure in pale green; the D232C-Kdo adduct in bright green; the wt-acceptor complex in pale yellow; and the D232C ternary complex in pale orange. Note that only very small local shifts are apparent in the protein structure between these states.

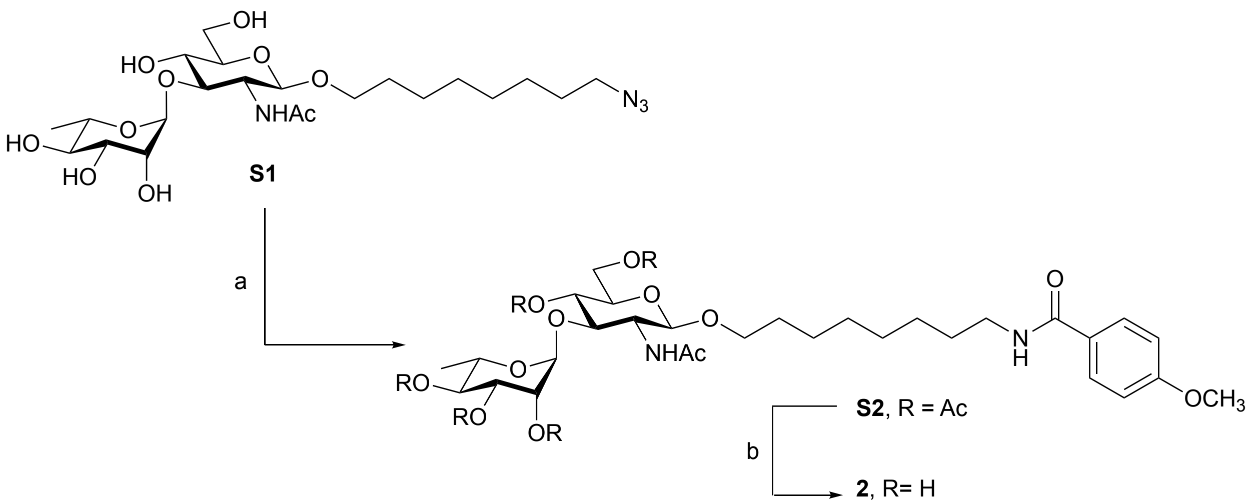

Supplementary Figure 11.
Acceptor **2** Synthesis. (a) triphenylphosphine, THF, rt, 48 h, then 4-methoxybenzoyl chloride, Et_3N, rt, 1 h, 71%; (b) CH_3ONa, CH_3OH, rt, 2 h, 96%.
The synthesis started from disaccharide 1a, which was prepared as described previously 10

8-(4-methoxybenzamido)octyl 2,3,4-tri-*O*-acetyl-α-L-rhamnopyranosyl-(1→3)-2-acetamido-4,6-di-*O*-acetyl-2-deoxy-β-D-glucopyranoside (2a). Disaccharide **1a** (53 mg, 0.07 mmol, 1.0 equiv) and triphenylphosphine (29 mg, 0.11 mmol, 1.5 equiv) were dissolved in THF (3.5 mL) and the resulting solution was stirred for 48 h. 4-Methoxybenzoyl chloride (25 mg, 0.15 mmol, 2.0 equiv) and triethylamine (2 drops) were added and the reaction mixture was stirred for 1 h. The reaction mixture was concentrated and the resulting residue was purified by column chromatography (CH_2Cl_2–EtOAc, 3:1 → CH_2Cl_2–EtOAc, 1:1) to afford disaccharide **2a** as a clear, yellow oil (45 mg, 71%): R_f = 0.28 (hexane–EtOAc; 2:3); $[\alpha]^{21}_D$+ 0.0 (*c* 0.82 $CHCl_3$); ^{1}H NMR (500 MHz, $CDCl_3$, δ_H) 7.73 (d, *J* = 9.0 Hz, 2 H, Ar), 6.92 (d, *J* = 9.0 Hz, 2 H, Ar), 6.22 (d, 1 H, *J* = 7.0 Hz, NH), 6.09 (t, 1 H, *J* = 5.5 Hz, NH), 5.22 (dd, 1 H, *J* = 2.4, 2.9 Hz, H-2′), 5.17 (dd, 1 H, *J* = 3.1, 10.4 Hz, H-3′), 5.15 (d, 1 H, *J* = 8.6 Hz, H-1), 5.04 (app t, 1 H, *J* = 10.1 Hz, H-4′), 4.98 (app t, 1 H, *J* = 10.1 Hz, H-4), 4.74 (d, 1 H, *J* = 2.1 Hz, H-1′), 4.55 (app t, 1 H, *J* = 9.7 Hz, H-3), 4.25 (dd, 1 H, *J* = 5.1, 12.3 Hz, H-6a), 4.07 (dd, 1 H, *J* = 2.5, 12.3 Hz, H-6b), 3.91–3.83 (m, 2 H, H-5′, octyl OCH_2), 3.85 (s, 3 H, OCH_3), 3.6 (ddd, 1 H, *J* = 2.4, 5.0, 10.2 Hz, H-5), 3.48 (dt, 1 H, *J* = 9.8, 6.6 Hz, octyl OCH_2), 3.43 (dt, 2 H, *J* = 2.0, 7.9 Hz, CH_2NH), 2.96 (app dt, 1 H, *J* = 10.2, 7.6 Hz, H-2), 2.11 (s, 3 H, CH_3CO), 2.08 (s, 6 H, CH_3CO x 2), 2.04 (s, 3 H, CH_3CO), 2.01 (s, 3 H, CH_3CO), 1.97 (s, 3 H, CH_3CO), 1.63–1.51 (m, 4 H, octyl CH_2 x 2), 1.41–1.30 (m, 8 H, octyl CH_2 x 4), 1.15 (d, 3 H, *J* = 5.8 Hz, H-6′); ^{13}C NMR (175 MHz, $CDCl_3$, δ_C) 171.7 (C=O), 170.8 (C=O), 170.5 (C=O), 170.2 (C=O), 170.0 (C=O), 169.7 (C=O),167.0 (C=O), 162.0 (Ar), 128.6 (Ar x 2), 127.2 (Ar), 113.7 (Ar x 2) 100.3 (C-1), 98.9 (C-1′), 80.3 (C-3), 71.5 (C-5), 70.4 (C-4), 70.3 (C-4′), 70.1 (octyl OCH_2), 69.5 (C-3′), 69.4 (C-2′), 67.9 (C-5′), 62.4 (C-6), 59.1 (C-2), 55.4 (OCH_3), 40.0 (CH_2NH), 29.7 (octyl CH_2), 29.4 (octyl CH_2), 29.1 (octyl CH_2 x 2), 26.8 (octyl CH_2), 25.7 (octyl CH_2), 23.5 (CH_3CO), 21.2 (CH_3CO), 20.9 (CH_3CO), 20.8 (C x 3, CH_3CO), 17.4 (C-6′); IR (cast film) 3288, 3076, 2931, 2932, 2857, 1747, 1661, 1634, 1607, 1547, 1506, 1438, 1371, 1297, 1224,

1180, 1119, 1076, 1046 cm^{-1}; HRMS (ESI): *m/z* calcd for $C_{40}H_{58}N_2O_{17}$ (M + Na) 861.3628, found 861.3618.

8-(4-methoxybenzamido)octyl α-L-rhamnopyranosyl-(1→3)-2-acetamido-2-deoxy-β-D-glucopyranoside (2) Disaccharide **2a** (36 mg, 0.04 mmol, 1.0 equiv) was dissolved in methanol (2.0 mL) and 1 M sodium methoxide (1 drop) was added. The reaction stirred for 2 h and then the sodium methoxide then quenched by the addition of prewashed Amberlite® IR 120 acidic resin before being filtered and concentrated. The residue was then dissolved in water and lyophilized to afford disaccharide **2** as a white solid (26.1 mg, 96 %). R_f = 0.60 (EtOAc–AcOH; 1:1); $[\alpha]^{21}_D$–47.1 (*c* 0.13, CH_3OH); ^{1}H NMR (500 MHz, CD_3OD, δ_H) 7.77 (d, 2 H, *J* = 9.0 Hz, Ar), 6.97 (d, 2 H, *J* = 9.0 Hz, Ar), 4.82 (d, 1 H, *J* = 1.3 Hz, H-1'), 4.40 (d, 1 H, *J* = 8.7 Hz, H-1), 3.93 (app dq, 1H, *J* = 9.6, 6.2 Hz, H-5'), 3.89–3.85 (m, 2H, H-6a, octyl OCH_2), 3.84 (s, 3H, $ArOCH_3$), 3.75 (dd, 1H, *J* = 1.3, 3.3 Hz, H-2'), 3.71 (app t, *J* = 8.6 Hz, H-2), 3.68 (dd, 1H, *J* = 5.3, 11.8 Hz, H-6b), 3.63 (dd, 1H, *J* = 3.2, 9.6 Hz, H-3'), 3.56 (app t, 1H, *J* = 9.0 Hz, H-3), 3.45 (dt, 1H, *J* = 9.5, 6.7 Hz, octyl OCH_2), 3.39–3.32 (m, 4H, H-4, H-4', CH_2NH), 3.26 (ddd, 1H, *J* = 2.2, 5.6, 9.6 Hz, H-5), 1.96 (s, 3 H, CH_3CO), 1.63–1.50 (m, 4 H, octyl CH_2 x 2), 1.40–1.31 (m, 8 H, octyl CH_2 x 4), 1.23 (d, 3H, *J* = 6.2 Hz, H-6'); C^{13} NMR (125 MHz, CD_3OD, δ_C): 173.3 (*C*=O), 169.8 (*C*=O), 163.8 (Ar) 130.0 (Ar x 2), 127.9 (Ar), 114.7 (Ar x 2), 103.3 (C-1'), 102.3 (C-1), 83.7 (C-3), 78.0 (C-5), 73.8 (C-4), 72.6 (C-2'), 72.2 (OCH_2), 70.8 (C-3'), 70.6 (C-5'), 70.4 (C-4'), 62.7 (C-6), 56.9 (C-2), 55.9 (OCH_3), 41.0 (CH_2NH), 30.6 (octyl CH_2 x 2), 30.4 (octyl CH_2), 30.3 (octyl CH_2), 28.0 (octyl CH_2), 27.0 (octyl CH_2), 23.0 (CH_3CO), 17.9 (C-6'); IR (cast film) 3313, 3093, 2932, 2856, 1635, 1608, 1506, 1442, 1376, 1311, 1257, 1180, 1050 cm^{-1}; HRMS (ESI): *m/z* calcd for $C_{30}H_{49}N_2O_{12}$ (M+Na) 651.3099, found 651.3095.

Supplementary Table 1. Theoretical and measured masses of detected by native MS $WbbB_{GT99}$-wildtype Hse species.

**$WbbB_{GT99}$-wildtype species**	**Formula**	**Theoretical Mass (Da)**	**Experimental Mass (Da)**
WbbBδM[a]	$C_{2073}H_{3140}N_{540}O_{617}S_{13}$	45915	45916 ± 1
WbbB	$C_{2078}H_{3149}N_{541}O_{618}S_{14}$	46046	46046 ± 1
WbbBδM*[a,b]	$C_{2073}H_{3142}N_{540}O_{616}S_{13}$	45901	45901 ± 1
WbbB* [b]	$C_{2078}H_{3151}N_{541}O_{617}S_{14}$	46032	46032 ± 1
WbbBδM*+Kdo[a,b]	$C_{2081}H_{3154}N_{540}O_{623}S_{13}$	46121	46121 ± 1
WbbB*+Kdo [b]	$C_{2086}H_{3163}N_{541}O_{624}S_{14}$	46252	46253 ± 1
$WbbB\delta M_2$[a]	$C_{4146}H_{6280}N_{1080}O_{1234}S_{26}$	91830	91832 ± 2
(WbbBδM+WbbB) [a]	$C_{4151}H_{6289}N_{1081}O_{1235}S_{27}$	91961	91960 ± 1
$WbbB_2$	$C_{4156}H_{6298}N_{1082}O_{1236}S_{28}$	92092	92091 ± 2
$WbbB\delta M_2$**+Kdo[a,c]	$C_{4154}H_{6296}N_{1080}O_{1239}S_{26}$	92023	92023 ± 1
(WbbBδM+WbbB)**+Kdo[a,c]	$C_{4159}H_{6305}N_{1081}O_{1240}S_{27}$	92154	92156 ± 2
$WbbB\delta M_2$**+CMP+Kdo[a,c]	$C_{4163}H_{6310}N_{1083}O_{1247}S_{26}P$	92346	92346 ± 1
(WbbBδM+WbbB)**+CMP+Kdo[a,c]	$C_{4168}H_{6319}N_{1084}O_{1248}S_{27}P$	92477	92477 ± 1
$WbbB\delta M_2$**+CMP+Kdo_2[a,c]	$C_{4171}H_{6322}N_{1083}O_{1254}S_{26}P$	92566	92566 ± 1
(WbbBδM+WbbB)**+CMP+Kdo_2[a,c]	$C_{4176}H_{6331}N_{1084}O_{1255}S_{27}P$	92697	92695 ± 2
$WbbB\delta M_2$**+$(CMP+Kdo)_2$[a,c]	$C_{4180}H_{6336}N_{1086}O_{1262}S_{26}P_2$	92889	92888 ± 1
(WbbBδM+WbbB)**+$(CMP+Kdo)_2$[a,c]	$C_{4185}H_{6345}N_{1087}O_{1263}S_{27}P_2$	93020	93020 ± 1

[a] WbbBδM designates protomers lacking the N-terminal Met (due to cleavage by Map during expression), WbbB designates protomers with N-terminal Met present. [b] * indicates that the molecular weight of the wildtype protomer species is shifted by 14 Da, as expected for a single Asp to Hse (homoserine) conversion. [c] ** indicates that molecular weight of WT dimer species

is shifted by 28 Da, corresponding to two Asp to Hse conversions. Note that, for dimeric species, either both WbbB protomers or neither protomer were converted to Hse; no species were observed with a single Hse conversion. This suggests that dimers that fail to react may have both protomers catalytically inactive, possibly as a consequence of freeze-thaw damage.

Supplementary Table 2. Assignment of the major Ms/MS fragments of the $(QVED(Hse)SNL+H)^+$ ion.

Fragment	*m/z* theoretical	*m/z* experimental	Fragment	*m/z* theoretical	*m/z* experimental
a_2-NH_3	183.11	183.11	b_5	573.25	573.25
a_2	200.14	200.14	b_6-H_2O	642.27	642.27
b_2-NH_3	211.11	211.11	b_6-NH_3	643.26	643.26
b_2	228.13	228.13	b_6/y_6- H_2O	660.28	660.28
y_2-NH_3	229.12	229.12	y_6-NH_3	661.27	221.27
y_2	246.14	246.14	y_6	678.29	678.29
y_3-NH_3	316.15	316.15	b_7-H_2O	756.32	756.31
y_3	333.18	333.18	b_7-NH_3	757.30	757.30
b_3-H_2O	339.17	339.17	b_7	774.32	774.32
b_3-NH_3	340.15	340.15	y_7	777.36	777.36
b_3	357.18	357.18	MH-H_2O	887.41	887.41
y_4-H_2O	416.21	416.21	MH-NH_3	888.39	888.39
y_4-NH_3	417.20	417.20	MH	905.42	905.42
y_4	434.22	434.22	ED(Hse)SN	547.20	547.20
b_4-H_2O	454.19	454.19	VED(Hse)	445.19	445.19
b_4-NH_3	455.18	455.18	ED(Hse)S	433.16	433.16
b_4	472.20	472.20	D(Hse)SN	418.16	418.16
y_5-H_2O	531.24	531.24	ED(Hse)	346.12	346.12
y_5-NH_3	532.22	532.22	D(Hse)S	304.11	304.11
y_5	549.25	549.25	(Hse)SN	303.13	303.13
b_5-H_2O	555.24	555.24	ED	245.08	245.08
b_5-NH_3	556.22	556.22	D(Hse)	217.08	217.08

[a]Hse = homoserine

Supplementary Table 3. Site directed mutagenesis primers

Mutation	Forward Primer	Reverse Primer
**R12A**	CTCCCTATCCGTTCGCGGGGCTTAAGGCCC	GGGCCTTAAGCCCCGCGAACGGATAGGGAG
**W20A**	GGGGGCTTAAGGCCCCTTATTTAGCGATGTTTTATAAATATCTTCAC	GTGAAGATATTTATAAAACATCGCTAAATAAGGGGCCTTAAGCCCCC
**W54A**	CTCAGCGCGCACGGGCGGAATTTGATCCGG	CCGGATCAAATTCCGCCCGTGCGCGCTGAG
**E158Q**	AGGTGATGCACATCCAGATTGGCCCGCTG	CAGCGGGCCAATCTGGATGTGCATCACCT
**R163A**	GAGATTGGCCCGCTGGCAGCACCAATGTATCG	CGATACATTGGTGCTGCCAGCGGGCCAATCTC
**D232C**	CGTGCTTCAGGTTGAGGATTGCTCCAATCTGATCGCCT	AGGCGATCAGATTGGAGCAATCCTCAACCTGAAGCACG
**D232N**	TTGAGGATAACTCCAATCTGATCGC	ATTGGAGTTATCCTCAACCTGAAGC

Supplementary Table 4. X-ray diffraction data collection and structure refinement statistics

	D232N+CMP-Kdo	D232N-Kdo	D232C-Kdo	wt+acceptor	D232C-ternary
RCSB I.D.	8CSB	8CSC	8CSD	8CSE	8CSF
**Data collection***					
Wavelength	0.97949	0.97950	0.97949	0.97950	0.97949
Space group	$C222_1$	$C222_1$	$C222_1$	$C222_1$	$C222_1$
Cell dimensions					
a, *b*, *c* (Å)	92.71, 157.44, 120.06	92.60, 158.22, 117.57	93.01 156.73 118.02	92.38, 158.02, 117.58	92.50, 158.07, 116.68
α, β, γ (°)	90, 90, 90	90, 90, 90	90, 90, 90	90, 90, 90	90, 90, 90
Resolution (Å)	48 – 2.25 (2.31 – 2.25)	40 – 1.90 (1.95 – 1.90)	47.5 – 1.95 (2.0 – 1.95)	50 - 2.3 (2.35 - 2.3)	46 – 2.4 (2.5 – 2.4)
R_{sym}	0.071 (0.74)	0.038 (0.965)	0.069 (0.81)	0.075 (0.78)	0.088 (0.96)
$I / \sigma I$	11.7 (2.0)	31.8 (2.07)	13.6 (2.05)	16.0 (2.2)	16.7 (2.11)
Completeness (%)	99.8 (99.7)	99.9 (99.9)	99.9 (100)	99.7 (99.9)	99.9 (100)
Redundancy	6.4 (6.7)	7.5 (7.5)	5.7 (5.8)	6.2 (6.0)	7.5 (7.5)
**Refinement**					
Resolution (Å)	48 - 2.25	38 - 1.9	47.5 - 1.95	37 - 2.3	46 - 2.4
No. reflections	41888	68059	62927	38435	33771
R_{work} / R_{free} (%)	20.1/24.4	19.4 /22.5	16.2/19.9	17.0/22.7	17.4/22.1
No. atoms					
Protein	6227	6330	6304	6313	6207
Ligand/ion	206	125	125	362	224
Water	85	416	357	305	250
B-factors					
Protein	76.4	50.6	45.8	48.7	51.1
Ligand/ion	75.3	40.6	34.3	53.9	42.0
Water	58.8	46.6	41.9	44.2	46.7
R.m.s. deviations					
Bond lengths (Å)	0.014	0.003	0.014	0.010	0.007
Bond angles (°)	1.29	0.50	1.02	1.08	0.89
Ramachandran plot					
Favored (%)	96.0	97.3	97.2	95.7	96.4
Allowed (%)	3.4	2.4	2.8	3.7	3.4

*Each dataset was collected from a single crystal. Values in parentheses are for the highest-resolution shell.
