# Supplementary material for: The retaining β-Kdo glycosyltransferase WbbB uses a double-displacement mechanism with an intermediate adduct rearrangement step
Source: Nat Commun. 2022 Oct 21;13:6277. doi: 10.1038/s41467-022-33988-1 (PMC9587256; doi:10.1038/s41467-022-33988-1)
Supplement: Supplementary file 2 — Description of Additional Supplementary files [file 41467_2022_33988_MOESM2_ESM.pdf]

Description of Additional Supplementary Files

File name: Supplementary movie 1
Description: Polder omit maps showing the ligands present in each active site.
